# Magnetosphere‐Ionosphere‐Thermosphere Coupling Study at Jupiter Based on Juno's First 30 Orbits and Modeling Tools

**DOI:** 10.1029/2022JA030586

**Published:** 2022-10-09

**Authors:** S. Al Saati, N. Clément, C. Louis, M. Blanc, Y. Wang, N. André, L. Lamy, B. Bonfond, B. Collet, F. Allegrini, S. Bolton, G. Clark, J. E. P. Connerney, J.‐C. Gérard, G. R. Gladstone, S. Kotsiaros, W. S. Kurth, B. Mauk

**Affiliations:** ^1^ IRAP CNRS Université Toulouse III‐Paul Sabatier CNES Toulouse France; ^2^ CPHT CNRS Institut Polytechnique de Paris Palaiseau France; ^3^ Laboratoire d’Astrophysique de Bordeaux Université de Bordeaux Bordeaux France; ^4^ School of Cosmic Physics DIAS Dunsink Observatory Dublin Institute for Advanced Studies Dublin Ireland; ^5^ LAM Pythéas Aix Marseille Université CNRS CNES Marseille France; ^6^ State Key Laboratory of Space Weather National Space Science Center Chinese Academy of Sciences Beijing China; ^7^ LESIA Observatoire de Paris Université PSL CNRS Sorbonne Université Université de Paris Meudon France; ^8^ University of Liège Liège Belgium; ^9^ SwRI San Antonio TX USA; ^10^ JHU‐APL Laurel MD USA; ^11^ NASA‐Goddard Space Flight Center Greenbelt MD USA; ^12^ Technical University of Denmark Kongens Lyngby Denmark; ^13^ University of Iowa Iowa IA USA

## Abstract

The dynamics of the Jovian magnetosphere is controlled by the interplay of the planet's fast rotation, its solar‐wind interaction and its main plasma source at the Io torus, mediated by coupling processes involving its magnetosphere, ionosphere, and thermosphere. At the ionospheric level, these processes can be characterized by a set of parameters including conductances, field‐aligned currents, horizontal currents, electric fields, transport of charged particles along field lines including the fluxes of electrons precipitating into the upper atmosphere which trigger auroral emissions, and the particle and Joule heating power dissipation rates into the upper atmosphere. Determination of these key parameters makes it possible to estimate the net transfer of momentum and energy between Jovian upper atmosphere and equatorial magnetosphere. A method based on a combined use of Juno multi‐instrument data and three modeling tools was developed by Wang et al. (2021, https://doi.org/10.1029/2021ja029469) and applied to an analysis of the first nine orbits to retrieve these parameters along Juno's magnetic footprint. We extend this method to the first 30 Juno science orbits and to both hemispheres. Our results reveal a large variability of these parameters from orbit to orbit and between the two hemispheres. They also show dominant trends. Southern current systems are consistent with the generation of a region of sub‐corotating ionospheric plasma flows, while both super‐corotating and sub‐corotating plasma flows are found in the north. These results are discussed in light of the previous space and ground‐based observations and currently available models of plasma convection and current systems, and their implications are assessed.

## Key Points

- We analyze the first 30 orbits of Juno to retrieve the properties of current systems and plasma flows associated with Jovian main auroras
- Southern hemisphere results are consistent with ionospheric plasma sub‐corotation
- The two opposite patterns, sub‐corotation and super‐corotation, are observed in the northern hemisphere

## 1 Introduction

### 1.1 Introduction to Jovian Magnetosphere and MIT Coupling

The Jovian magnetosphere stands out as an archetype of giant planet magnetospheres. Its dynamics is driven by three main sources of mass, momentum and energy: planetary rotation, which is enforced to the magnetosphere via its coupling with the conducting ionospheric layer of the upper atmosphere; the solar wind, which is coupled to the magnetosphere via different boundary layers and a partial interconnection of solar wind and magnetospheric field lines; and planetary moons, which display a broad variety of interactions with the planet's magnetic field, plasma flows and energetic particles involving their interiors, surfaces and exospheres/ionospheres.

These exchanges of mass, momentum and energy between the planet, its moons and the solar wind are mediated by coupling processes involving transmission of electromagnetic fields and waves, flows of electric currents and transport of particles mainly along magnetic field lines. These processes occur between three regions: the equatorial magnetosphere where the magnetodisk and moons reside, the high‐latitude polar and auroral magnetosphere through which most of these exchange processes take place, and the Jovian ionosphere/thermosphere, referred to later as Regions I, II, and III following Wang et al. (2021). Electric current loops play an important role in these processes. In the quasi‐static approximation, the divergence of electric currents flowing across magnetic field lines in the magnetodisk and moons' plasma envelopes is balanced by currents flowing along magnetic field lines, closing the circuit in the planet's ionospheric conductor. The resulting direct current loops transfer momentum between the equatorial magnetosphere, the solar wind and the upper atmosphere via the associated Lorentz force (as described for instance by Cowley & Bunce, 2001; Cowley et al., 2005; Nichols, 2011; Nichols & Cowley, 2005; Ray et al., 2010, 2012; Southwood and Kivelson, 2001). At the ionospheric end of these current loops, Joule heating by these currents adds to charged particle precipitation to dissipate power into the upper atmosphere, setting it into large‐scale motion, as described by Achilleos et al. (1998); Bougher (2005); Tao et al. (2009); Smith and Aylward (2009); Ray et al. (2015). Propagation of Alfvén waves between magnetosphere and ionosphere also plays an important role, not yet fully understood, in these exchanges of particles, momentum and energy along magnetic field lines (see Saur et al., 2018).

### 1.2 Auroral Emissions and Particle Precipitation

Auroral emissions and their causes via particle precipitation into the upper atmosphere are an important manifestation of Magnetosphere‐Ionosphere‐Thermosphere coupling processes, referred to later as “MIT coupling processes.” Before and during the advent of in situ exploration by Jupiter flybys or orbiters (Voyager, Galileo, Juno), MIT coupling processes could only be probed remotely from the observations of electromagnetic emissions produced in the auroral and polar regions at radio, optical (UV, visible, IR) and X‐ray wavelengths (Clarke et al., 2004). With the advent of Juno direct observations over the auroral and polar regions, these emissions could be related more directly to particle precipitation patterns, as we are going to review briefly here.

X‐ray emissions were observed using the Einstein observatory (Metzger et al., 1983), Röntgen satellite (Waite et al., 1994) and later Chandra (Gladstone et al., 1998) and X‐ray Multi‐Mirror Mission (see Wibisono et al., 2020, and references therein).

UV auroral emissions, most extensively studied so far, are emissions of H (Ly‐*α*) and $H_{2}$ (in the Werner and Lyman bands) mainly triggered by the precipitation of energetic electrons into the upper atmosphere, of which they are a direct tracer. Hubble Space Telescope (HST) high resolution images of the UV aurorae of Jupiter regularly acquired since the late 1990s (Clarke et al., 1998; Gérard et al., 2014, and references therein) have revealed that these aurorae mainly consist of three main components with approximately equal contributions to the total emitted power (Grodent et al., 2018). Following an order of increasing latitude, one finds first (a) the outer emissions located on the equator side of the main oval, corresponding to diffuse, patchy and sometimes arc‐like auroral emissions, and containing among them the Galilean moons magnetic footprints. Then, one finds (b) the relatively stable main auroral oval, and finally (c) the variable polar emissions distributed irregularly across the polar cap. This average auroral morphology is further complicated by some finer structures (e.g., Grodent, 2015, and references therein). This distinction between these three components ((a) diffuse emissions on the equatorial side of the main ovals, (b) the main oval and (c) polar emissions) used to be associated with the three magnetospheric momentum sources mentioned in our introduction (i.e., moons interactions, planetary rotation and coupling to the solar wind), but recent observations, particularly by Juno, suggest that the separation of these three sources in the generation of auroral emissions is significantly more complex and still poorly understood (e.g., Bonfond et al., 2020).

IR auroral emissions (see review by Miller et al., 2020) are mainly produced by thermally excited rovibrational transitions in the 3–4 microns domain of the ${H_{3}}^{+}$ radical, which is the main constituent of the Jovian ionosphere in the altitude range where ionospheric conductances peak. ${H_{3}}^{+}$ is created through two reactions, beginning with ionisation of molecular hydrogen mainly caused by electron precipitation in the auroral regions. The ${H_{2}}^{+}$ ion then quickly reacts with molecular hydrogen to produce ${H_{3}}^{+}$. As ${H_{3}}^{+}$ emissions are thermal, different rovibrational transitions are achieved at different temperatures. The total line intensity seen from a specific direction is proportional to the total content of the ${H_{3}}^{+}$ emitting ions along the line of sight. The Doppler shift of emission lines can be used to derive the ${H_{3}}^{+}$ line‐of‐sight velocity, from which the ionospheric flows can be inferred. IR auroral observations were obtained mainly from ground‐based telescopes (Drossart et al., 1989; Stallard et al., 2003, 2018), using CSHELL (Cryogenic Near‐IR Facility Spectrograph) at the NASA Infrared Observing Facility (IRTF) in Hawaii (Stallard et al., 2003), the CRIRES (Cryogenic high‐resolution infrared echelle spectrograph) instrument at the ESO Very Large Telescope (VLT) in Chile (Johnson et al., 2017, 2018), and the NIRSPEC instrument on Keck II (O’Donoghue et al., 2021; Wang et al., 2022) to study ${H_{3}}^{+}$ plasma flows over the auroral and polar regions. Johnson et al. (2016) used the IRTF to show that, in contrast to high latitudes, the middle and low latitude thermosphere rotates rigidly with the planet, thus indicating that departures from corotation are a specific feature of high latitudes. ${H_{3}}^{+}$ can also be used to trace heating sources of the thermosphere: see the recent study by O’Donoghue et al. (2021) using the NIRSPEC instrument on the Keck II telescope, which strongly suggests that polar and auroral sources are dominant in global heating of the thermosphere even at middle and low latitudes.

From the early observations by Juno using the JIRAM spectro‐imager, the same three regions of auroral emissions are seen in IR as in UV, with similarities and differences to UV emissions which inform us about the processes controlling these emissions (e.g., Adriani et al., 2017, and references therein).

Jovian auroral radio emissions were first observed by Burke and Franklin (1955). They used to be described as a superposition of three components corresponding to three partly overlapping frequency bands: broadband kilometric (∼10–400 kHz), hectometric (HOM, 250–3 MHz) and decametric (DAM, 3–∼40 MHz) emissions. The sources of these emissions, which are produced along auroral field lines near the local electron gyrofrequency, extend from the topside ionosphere to a few planetary radii. The DAM component is primarily controlled by the interaction between Jupiter and the Galilean moon Io (Bigg, 1964), with additional contributions from Europa and Ganymede (Louis et al., 2017; Zarka et al., 2017, 2018). Juno radio, magnetic field, and electron in situ measurements within the emission region of these various components have confirmed that these radio waves are powered by the Cyclotron Maser Instability (CMI), as is the case at Earth and Saturn. The CMI appears to be mainly driven by electrons of few keV energy with a loss‐cone distribution function. The statistical analysis of the KOM/HOM and (non‐satellite) DAM radiosources crossed by Juno revealed that they are spatially colocated along the same field lines, which map to the equatorward portion of the main auroral oval (Imai et al., 2019; Louis et al., 2019). In particular, DAM emissions are observed above the UV emission just equatorward of the brightest UV emission of the main oval (Louis et al., 2019; Wang et al., 2021).

Prior to the arrival of Juno at Jupiter, while it was in the solar wind upstream of the planet, combined observations of the Jovian UV aurora by HST (Nichols et al., 2017) and of the IR aurora by NIRSPEC on Keck II (Moore et al., 2017) were performed, using Juno plasma and magnetic field instruments as a solar wind monitor. They could reveal the passage of several interplanetary shocks and their corresponding effects on Jovian auroras, providing some indication on the respective roles of the solar wind and of internal processes in the temporal variation of these auroras.

Once inside the magnetosphere, Juno provided direct in situ access to the high latitude magnetosphere and topside ionosphere along its polar low‐periapse orbits. Since then, in situ measurements of magnetic fields, charged particles and waves, together with radio, UV and IR remote sensing observations of auroral emissions conjugate to the spacecraft opened the possibility of directly determining the variations of several of the MIT coupling parameters across and around the main oval. Field‐aligned currents flowing between the ionosphere and magnetosphere were determined by Kotsiaros et al. (2019). The variations of electron fluxes along field lines determined by JADE (Allegrini et al., 2017, 2020) and JEDI (Mauk et al., 2013) instruments have been extensively analyzed. In particular, Mauk et al. (2020) were able to show the existence of different regimes of electron precipitation as a function of latitude. In order of increasing latitude, they identified a regime of diffuse precipitation prevailing equatorward of the main oval, called “difA,” characterized by electron populations with electron intensities outside of the loss cone larger than the intensities inside the loss cone, and downward intensities and energy fluxes greater than the upward intensities and energy fluxes. Then they identified the Region I precipitation regime, dominated by inverted‐V structures and mono‐directional downward acceleration of electrons inside the loss cone and prevailing over a large fraction of the main oval. Finally, they identified the Region II precipitation regime, over a fraction of the main oval and poleward of it, often dominated by bi‐directional acceleration of electrons. If Juno is below the acceleration region, the net energy deposition resulting from downward precipitation becomes directly accessible from the JADE and JEDI data. While the “difA” region tends to be located equatorward of the UV main oval, region I and II precipitation regimes overlap with the main UV oval.

### 1.3 Multi‐Instrument Description of MIT Coupling Parameters at Ionospheric Altitudes

Auroral emissions, mainly caused by particle precipitation into the upper atmosphere, are only one manifestation of MIT coupling processes at ionospheric altitudes. Taking a broader perspective, these processes can be characterized by a set of “MIT coupling key parameters,” as described in Section 1.2 of Wang et al. (2021), its Table 1 and Figure 2. Determination of these parameters allows one to estimate the net transfer of momentum and energy between the Jovian upper atmosphere and the equatorial magnetosphere, as well as some components of the exchange of charged particles between ionosphere and magnetosphere along field lines. Indeed, particle exchanges between the Jovian upper atmosphere and the equatorial magnetosphere can be described by the transport of charged particles along field lines including the fluxes of electrons precipitating into the upper atmosphere which trigger auroral emissions. These transport parameters consist in a first set of relevant coupling parameters. Then, the net exchange of momentum can be characterized via the parameters describing current closure in the ionosphere such as the ionospheric conductances *Σ*_*H*_ (Hall), *Σ*_*P*_ (Pedersen), the field‐aligned currents *J*_‖_, the horizontal ionospheric currents *J*_iono_, and the ionospheric electric fields *E*_iono_. Finally, energy deposition into the upper atmosphere can be characterized by two parameters being the particle power dissipation rate into the upper atmosphere *P*_*e*_ and the Joule heating rate of the upper atmosphere *P*_*J*_.

Wang et al. (2021) described how several of these MIT coupling parameters, including ionospheric conductances, electric currents, electric fields and Joule heating, which are not directly accessible by a single Juno instrument, can be derived from a combination of Juno instruments, using simple adequate models based on Maxwell's equations and the laws of ionospheric electrodynamics. Determination of this set of MIT coupling parameters along the Juno magnetic footprint and across the main oval is possible as long as variations of all parameters along this oval can be regarded as much smaller than variations across the oval over similar distances. Wang et al. (2021) applied this method to the first nine southern polar cap fly bys of the Juno mission and analyzed in detail two southern oval crossings during perijoves 3 and 6. This initial study made it possible to provide a picture of the Magnetosphere‐Ionosphere‐Thermosphere coupling current systems. The two southern crossings studied in detail showed the existence of a pair of field‐aligned currents: a first upward current coincident with the largest downward electron fluxes and a downward current poleward of it, connected by horizontal equatorward ionospheric Pedersen currents. Both this current system and the associated equatorward electric fields and westward drifts (opposite to corotation) were found to be reasonably consistent with predictions of the corotation‐enforcement current model of Cowley et al. (2005, 2008, 2017). The amount of angular momentum and power extracted from the ionosphere to the magnetodisk by the calculated current systems, as well as the total power dissipated into the thermosphere via Joule heating and electron precipitation, were also estimated. Finally, the E × B drift velocities derived from Juno multi‐instrument data by Wang et al. (2021) could be compared with horizontal ionospheric E × B plasma drifts derived from Earth‐based telescopic observations of the Doppler shift of Jovian ${H_{3}}^{+}$ IR emissions (Johnson et al., 2017, 2018; Stallard, 2001).

A statistical study of these first eight southern crossings also showed that the auroral Pedersen and Hall conductances are significantly enhanced by electron precipitations and vary from less than 1 mho to over 10 mhos. These results could be compared to previous studies of ionospheric conductivity enhancements by Nichols and Cowley (2004); Ray et al. (2010, 2012), and Gérard et al. (2020). They also showed that Hall conductances are usually twice as large as the Pedersen ones. Joule heating near the main oval can be as large as ∼1.0 W/m^2^, and westward ionospheric plasma flows of fractions of a km/s were observed, in fair agreement with subcorotating flows predicted in the corotation enforcement models of Cowley and Bunce (2001) and Cowley et al. (2003).

While the preliminary study of Wang et al. (2021) made it possible to design and test a method for the derivation of MIT coupling parameters from Juno observations, it was limited to the 9 first science orbits and only to observations of the southern hemisphere.

### 1.4 Objective of This Study

The main objective of this new study is to provide a description of MIT coupling processes, as seen at ionospheric altitudes from Juno, that encompasses both the northern and southern main auroral ovals and to capture the main trends of MIT coupling at the level of the main auroras in terms of the associated coupling current loops, particle precipitation patterns, energy deposition rates into the upper atmosphere, and their mutual relationships, over a broad local time range.

To this end, we extend the method of Wang et al. (2021), with some improvements that will be described, to the thirty first crossings of the northern and southern main ovals by Juno. This significant extension of the analyzed data set, which covers most of the Juno nominal mission, allows us to capture more systematically both the dominant trends and the variability, inter‐hemispheric and from orbit to orbit, of MIT coupling parameter variations across the main ovals. It gives us access to a broader range of local time thanks to the azimuthal drift of Juno's orbit with time. As we will see, it also provides some clues to the separate modes and degree of coupling of the northern and southern main oval regions to the magnetosphere and its magnetodisk, and on the consistency of the corotation enforcement model with data from the two hemispheres.

## 2 Methodology, Models and Data Handling

The method initially developed by Wang et al. (2021) and improved on several aspects in this study relies on an interplay between Juno data inputs and three models in the flow of calculations that leads to the derivation of estimates of the different MIT coupling parameters at ionospheric altitudes. We first briefly describe the three models, before presenting the flow of calculations that produces these estimates.

### 2.1 Overview of the Models

The three models used have been extensively described in appendices A‐1 (for the atmosphere model), A‐2 (for the ionosphere model) and A‐3 (electrodynamics model) of the Supplementary Information of Wang et al. (2021). The improved version of the electrodynamics model used for this study is also presented in the Supplementary Information of the present article. We summarize their main characteristics here.

#### 2.1.1 Atmosphere Model

The Jovian atmospheric model considered here has been adapted to Juno studies by Wang et al. (2021) from the Jupiter Transplanet Atmosphere Model (Blelly et al., 2019), which is a semi empirical model using the assumptions of diffusive equilibrium well above the homopause and of mixed equilibrium well below the homopause. Its theoretical foundations are described in Banks and Kockarts (1973). This model describes the temperature and vertical distribution of the different thermospheric neutral species ($H,\;H_{2},\;\mathrm{He},\;{\mathrm{CH}}_{4},\;C_{2}H_{2}$) with the help of 17 free parameters that can be adjusted to observational data from Juno and possibly other sources.

#### 2.1.2 Ionosphere Model

Our Jovian ionosphere model calculates the altitude distribution of the ionization rate for specified energy‐dependent electron precipitation spectra and for a given neutral atmosphere vertical structure obtained from the atmospheric model. It assumes local chemical equilibrium of the Jovian ionosphere, following Hiraki and Tao (2008) and Gérard et al. (2020), and uses JADE and JEDI combined electron precipitation spectra to provide estimates of the altitude distributions of ion and electron densities. It derives from them the vertical profiles of Pedersen and Hall conductivities and eventually their height‐integrated values, namely the Pedersen and Hall conductances. Finally, it also computes the particle heating rate corresponding to the downward electron energy flux in the loss cone measured by the particle detectors.

This model neglects ionization sources other than electron precipitation, such as solar photons and also the meteoric ion contribution, which Nakamura et al. (2022) recently showed to be significant at low and middle latitudes. For auroral conditions, however, the meteoric contribution to ionospheric conductances found by Nakamura et al. (2022) is on the order of a few tenths of mhos, negligible with respect to the conductances of several (up to 10) mhos generated by electron precipitation, as the results of the present study show.

#### 2.1.3 Electrodynamics Model

**Figure 1:**
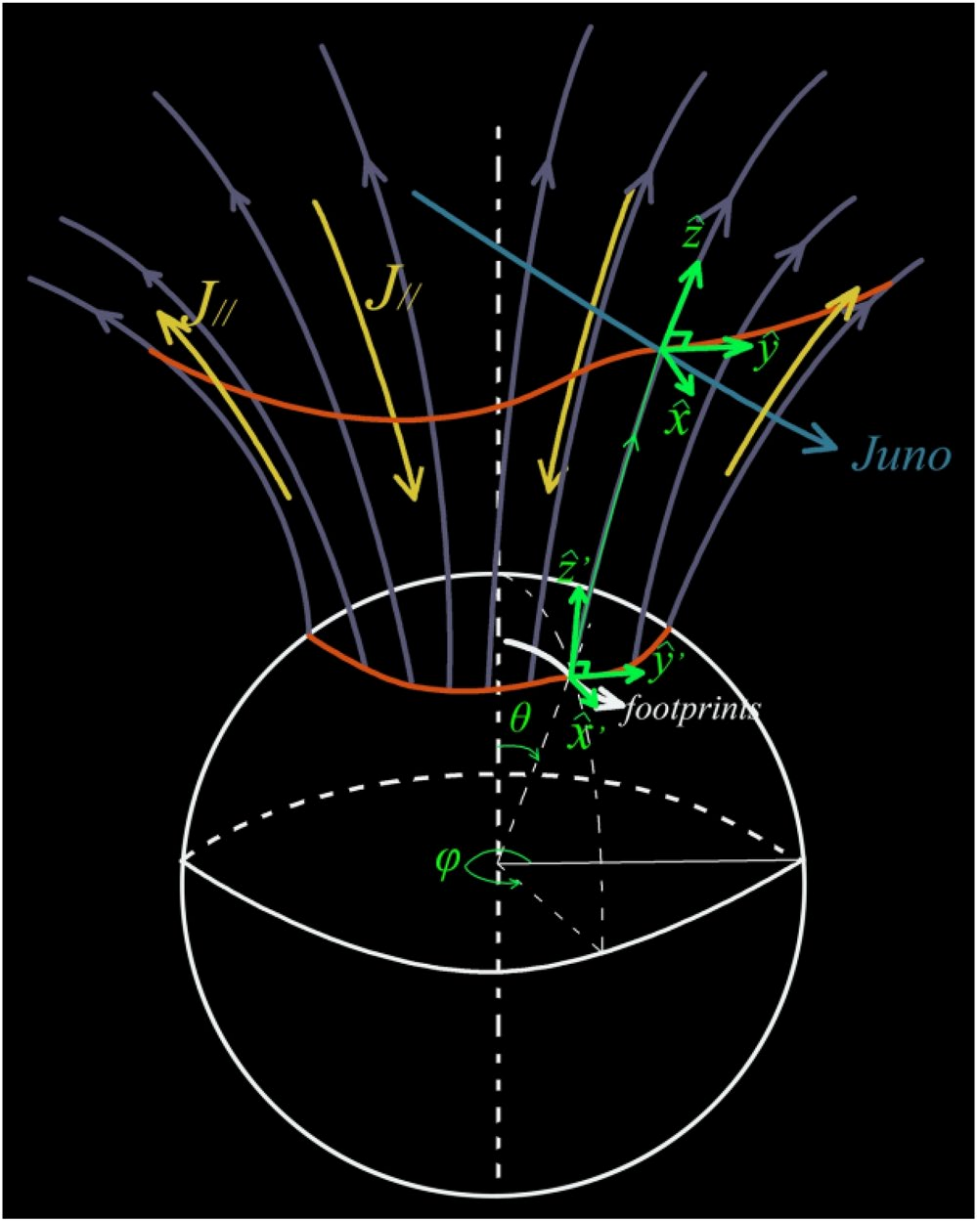
Illustration of geometric elements and reference frames used in our calculations of Magnetosphere‐Ionosphere‐Thermosphere (MIT) coupling parameters from Juno data, here for the northern hemisphere. The mean location of the main oval is shown as the lower red curve on the ionospheric sphere. The magnetic field lines mapping to this mean location are shown in purple. They make it possible to map this main oval location to the altitude of Juno, shown as the upper red curve. Juno's trajectory as well as the trajectory of its magnetic footprint are shown in sky‐blue and white, respectively. Two local reference frames are used to perform the differential calculations based on Maxwell's equations from which we derive the MIT coupling parameters: (a) At the altitude of Juno and everywhere along magnetic field lines above the ionosphere (Region II), unit vector $\hat{z}$ points along the magnetic field, $\hat{y}$ is positive eastward along the tangent to the cone of field lines connected to the main oval, and $\hat{x}$ complements the frame, positive toward the equator in both hemispheres; (b) at the ionospheric altitude (Region III), unit vectors ${\hat{x}}^{\prime }$ and ${\hat{y}}^{\prime }$ are horizontal and tangent to the conducting layer of the ionosphere which is assumed to be an infinitely thin surface surrounding the plane. ${\hat{x}}^{\prime }$ is oriented toward the equator orthogonal to the auroral oval (red curve), ${\hat{y}}^{\prime }$ is oriented eastward along the oval, and ${\hat{z}}^{\prime }$ is vertical, positive upward in the northern hemisphere and downward in the southern hemisphere. ${\hat{x}}^{\prime }$ and ${\hat{y}}^{\prime }$ taken together provide a local 2‐D reference frame for horizontal vectors and differential expressions defined in the plane tangent to the ionospheric conducting layer. Figure from Supplementary Information of Wang et al. (2021).

The electrodynamics model calculates field‐aligned currents, ionospheric electric currents, electric fields and Joule heating from two sources: magnetic field variations measured along Juno orbit by the MAG instrument, and Hall and Pedersen conductances delivered by the ionosphere model. To do so it performs a geometric mapping along magnetic field lines of the parameters that are measured by Juno along its trajectory on high‐altitude auroral and polar field lines (Region II in Wang et al., 2021) down to the conducting layer of the ionosphere/thermosphere (Region III in Wang et al., 2021). This conducting layer is modeled as a 2‐D infinitely thin shell surrounding the planet, since its vertical thickness is only on the order of $3\times 10^{-3}$ times the Jovian radius R_J_. To cover Region II, Region III and their magnetic connections, we use two different local reference frames just as in Wang et al. (2021), and as described in Figure 1.

At the altitude of Juno, in Region II, the local reference frame $\left(\hat{x},\hat{y},\hat{z}\right)$ is defined such that $\hat{z}$ points along the magnetic field, $\hat{y}$ is positive eastward along the tangent to the cone of field lines connected to the main oval, and $\hat{x}$ complements the frame. At the level of the ionosphere, ${\hat{x}}^{\prime }$ and ${\hat{y}}^{\prime }$ are horizontal and tangent to the conducting layer of the ionosphere. ${\hat{x}}^{\prime }$ is oriented toward the equator orthogonal to the auroral oval, ${\hat{y}}^{\prime }$ is oriented eastward along the oval, and ${\hat{z}}^{\prime }$ is vertical, positive upward in the northern hemisphere and downward in the southern hemisphere.

Given the 1‐D sampling of space provided by Juno's trajectory, calculation of MIT coupling parameters is possible only by assuming that the variations of all quantities along the main auroral oval are much smaller than variations orthogonal to the oval and to the local direction of the magnetic field. This condition consists in imposing $\frac{\partial }{\partial x}\gg \frac{\partial }{\partial y}$ in Region II, and $\frac{\partial }{\partial x^{\prime }}\gg \frac{\partial }{\partial y^{\prime }}$ in Region III. It allows one to reduce the different Maxwell equations used in our calculations to one‐dimensional differential equations with respect to the x and $x^{\prime }$ variables.

**Figure 2:**
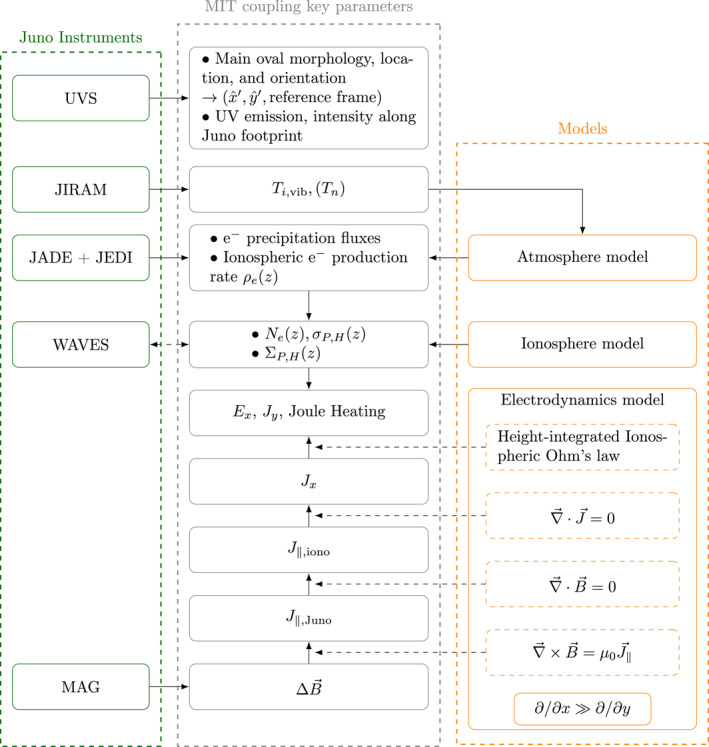
Calculation scheme used to retrieve the key parameters of Magnetosphere‐Ionosphere‐Thermosphere coupling (gray boxes) from Juno data (green boxes) and three models (orange boxes). This figure depicts the flow of information (plain arrow) and computation (dashed arrow) from the data and from the models used to compute the coupling parameters.

The calculation flow goes as follows (see also Figure 2): first, the local field‐aligned current flowing at the location of Juno, *J*_‖,Juno_, is deduced from magnetic field variations along its trajectory using the $\vec{\nabla }\times \vec{B}=\mu_{0}\vec{J}$ equation. Then we use the $\vec{\nabla }\cdot \vec{B}=0$ equation, which can be reduced to $\frac{J_{\|,Juno}}{B_{Juno}}=\frac{J_{\|,iono}}{B_{iono}}$ , to deduce the field‐aligned current at the top of the ionosphere, *J*_‖,iono_, from the one calculated at Juno's altitude. Then, the horizontal height‐integrated ionospheric current *J*_*x*_ orthogonal to the local direction of the main oval is deduced from the integration of $\vec{\nabla }\cdot \vec{J}=0$ across the thickness of the ionospheric conductor.

In the final step of calculations, the model uses the ionospheric Ohm's law in its height‐integrated form and ionospheric conductances delivered by the ionosphere model

$$\vec{J}=\bar{\bar{\Sigma }}\vec{E}+\int_{z}\bar{\bar{\sigma }}\left(\vec{u}\times \vec{E}\right)dz$$

where *u*_*x*_(*z*) and *u*_*y*_(*z*) are the neutral wind components respectively along and orthogonal to the local direction of the main oval, to calculate the second component *J*_*y*_ of the electric current flowing along the direction of the main oval, the *E*_*x*_ component of the electric field (neglecting the neutral wind terms in the ionospheric Ohm's law) and the height‐integrated Joule heating of the thermosphere. Rigorously, as shown in appendix A‐3 of the Supplementary Information of Wang et al. (2021), when the model calculates the electric field orthogonal to the main oval by dividing the horizontal current *J*_*x*_ by the Pedersen conductance, it finds an average electric field corrected by the dynamo effects of the neutral winds along ($u_{x}$) and orthogonal ($u_{y}$) to the local direction of the main oval, which can be written as

$$E_{x}^{\prime }=E_{x}+B_{z}{\langle u_{y}\rangle }_{\text{Pedersen}}-\frac{\Sigma_{P}}{\Sigma_{H}}{\langle u_{x}\rangle }_{\text{Hall}}$$

In this equation, the two neutral wind terms, which are weighted averages of the vertical distributions of the neutral wind respectively along and orthogonal to the main oval taking the Pedersen and Hall conductivities respectively as weighting factors, cannot be directly deduced from our scheme, but their magnitude relative to *E*_*x*_ can be evaluated from models. For the typical electron precipitation energies of 10–100 keV observed in the traversals of the main oval by Juno, conductivities reach their largest values over a limited altitude range, between 260 and 360 km of altitude for the Pedersen conductivity, and between 220 and 280 km of altitude for the Hall conductivity (see Figure A2‐1 of Wang et al., 2021). Several models of the response of the Jovian neutral atmosphere to auroral energy deposition can be inspected to assess the amplitude of the neutral wind terms: Bougher (2005), Tao et al. (2009, see their Figure 3), Smith and Aylward (2009), and Ray et al. (2015) ‐ see Figure 5 of Ray et al. (2015) which compares the result of their model, on the right‐hand side, with the results of Smith and Aylward (2009) on the left‐hand site. Neutral winds driven by auroral energy deposition appear to be small (on the order of a few tens of m/s) in the altitude ranges where conductivities are significant. They reach larger speeds of hundreds of m/s or more at significantly larger altitudes of 400 km and above. This implies that, according to current models of thermospheric response to auroral heating, neutral winds can be neglected in our calculation. One can also note that field line slippage is not relevant for the conditions of our Juno observations: field lines are anchored into the ionospheric conductor significantly below the altitudes where ion drag is large enough to drive the much denser neutral atmosphere into motion. The same reasoning applies to the Joule heating term, which is calculated from the product of *J*_*x*_ and *E*_*x*_ neglecting a similarly small neutral wind term (see the Supplementary Information of Wang et al., 2021, for a more detailed analysis of this issue).

In addition to the two local coordinates, the global reference frame used in this study is Jupiter's magnetic dipole coordinate system linked to the dipole component of the JRM09 model. In most of the cases considered in this study, the azimuthal direction in this global reference frame was locally approximately coincident with the direction tangent to the main oval. We thus approximated most of the time the coordinates $x^{\prime }$ and $y^{\prime }$ to be respectively the *θ* and *ϕ* coordinates of the global magnetic reference frame. This condition, which simplifies the calculation scheme, is however not a necessary condition for the validity of our calculations, which can be performed in the two local reference frames independently of the choice of the global reference frame, provided that appropriate magnetic mapping between them is secured.

We refer the reader to the Supplementary Information of this present study and of Wang et al. (2021) for a more comprehensive description of these three models.

### 2.2 Calculation Scheme

The calculation scheme elaborated by Wang et al. (2021) and improved on some aspects in this study is illustrated in Figure 2, which is a different representation of Figure 4 of Wang et al. (2021) allowing a closer description of the flow of information and computations (shown by arrows) from Juno data (left‐hand side column) and from models (right‐hand side column) used to calculate the different MIT coupling parameters (central column). One can see that information flows mainly from the top and from the bottom of the figure.

From the bottom, subtraction of an average magnetic field model (JRM09 magnetic field model from Connerney et al. (2018) + CAN81 current sheet model from Connerney et al. (1981)) from data provided by the MAG investigation (Connerney et al., 2017) provides a residual magnetic field. This residual magnetic field is used to calculate field‐aligned currents at Juno altitudes, then at ionospheric altitudes, and finally horizontal ionospheric currents orthogonal to the main oval, using a 1‐D reduction of Maxwell's equations as explained in our short introduction to the electrodynamics model given above.

From the top, the UVS spectro‐imaging instrument (Gladstone et al., 2014) is used first to study the morphology and orientation of the main oval and the configuration of the trajectory of Juno relatively to it, and to identify the time periods during which the crossings of the main oval by Juno's magnetic footprint occur. The JIRAM spectro‐imaging instrument (Adriani et al., 2014) can then be used to provide an estimation of the vibrational temperature of the $H_{3}^{+}$ dominant ionospheric ion, and of the neutral temperature of the lower thermosphere under the assumption of local thermodynamic equilibrium, to feed our atmosphere model. However this can be done only when simultaneous observations of the same segment of the main oval by UVS and JIRAM are available.

Then, using JADE (McComas et al., 2013) and JEDI (Mauk et al., 2013) data, continuous pitch angle and energy distributions of electron fluxes from 100 eV to 2 MeV are obtained, together with the total electron energy deposition into the thermosphere. These parameters are used to retrieve the values of the ionospheric Pedersen and Hall conductivities and conductances from the ionosphere model.

At the convergence point of upward and downward flows of information in Figure 2, entering ionospheric currents (produced by the electrodynamics model) and conductances (produced by the ionosphere model) into the height‐integrated ionospheric Ohm's law makes it possible to calculate the component *J*_*y*_ of the ionospheric current flowing along the main oval, the horizontal ionospheric electric field (with a correction for the effect of neutral winds averaged across the ionospheric conducting layer ‐ see description of the electrodynamics model above) and an estimate of Joule heating.

Finally, radio and plasma waves data provided by WAVES (Kurth, Hospodarsky, et al., 2017​) are used to study the radio emissions co‐located with Juno during its traversal of auroral zone field lines.

The method used to derive a residual magnetic field representing the local effect of field‐aligned currents from MAG data is an important aspect of this procedure. In Wang et al. (2021), a baseline variation was adjusted to the difference between observed and model fields to produce the final residual field for each particular fly by of the main oval. In this study, in order to design an automated process, we used Fast Fourier Transform filtering methods to limit the residual magnetic field to the frequency band assumed to be relevant for our study, which is determined for each crossing. This residual magnetic field $\delta \vec{B}$ is defined as $\delta \vec{B}={\vec{B}}_{\mathrm{MAG}}-{\vec{B}}_{JRM09+CAN81}$ where ${\vec{B}}_{\mathrm{MAG}}$ is the magnetic field measured by Juno's MAG instrument, and ${\vec{B}}_{JRM09+CAN81}$ is the magnetic field obtained from the JRM09 + CAN81 magnetic field model. It includes both fluctuations at spatial scales larger than the typical scale of the main oval crossing, and residual small‐scale fluctuations at Juno's spinning period of 30 s and higher frequencies. Both of these low‐frequency and high‐frequency components are assumed to be irrelevant for the analysis of the field‐aligned currents associated to the main oval, which are expected to show up at spatial scales on the order of its thickness. To remove them, we applied a band‐pass filter to the residual magnetic field data, with a frequency band tailored to each crossing depending on the scale of the auroral oval. One end of the band cuts out small‐scale fluctuations, and the other end cuts out currents at much larger spatial scales than the oval. The effects of this band‐pass filter on the retrieved residual field and on the significance or our results are discussed in detail in the Supplementary Information.

## 3 Results From Juno's First 30 Orbits

The orbit of Juno is a highly eccentric polar ellipse with a period of 53 days, with an apojove radius of ∼100 R_J_ and a perijove radius of ∼1.05 R_J_. This orbit allows Juno to successively fly by the northern polar cap, to next reach its perijove, to finally fly by to southern polar cap before leaving the vicinity of Jupiter in a very condensed amount of time of a few hours. This complete sequence where Juno is close to Jupiter is referred to as “perijove” in the literature, as a shorthand notation to describe this short duration sequence of high scientific interest, and we will use this term in the following.

### 3.1 Description of the Data Set

**Figure 3:**
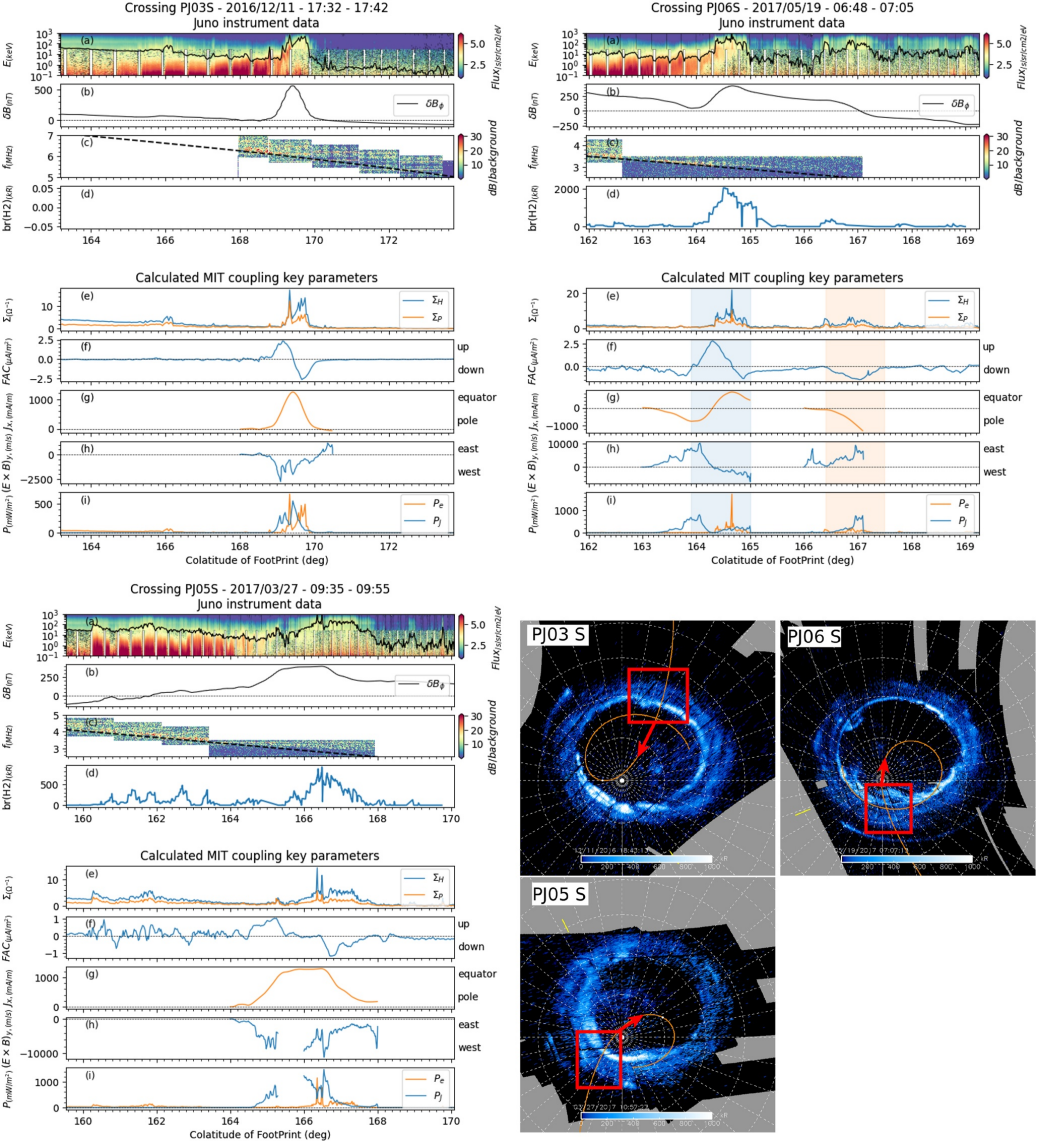
Juno instrument data (top four rows of each panel) and Magnetosphere‐Ionosphere‐Thermosphere (MIT) key parameters calculated from them (bottom five rows of each panel) plotted as a function of the magnetic colatitude of Juno's magnetic footprint for southern perijoves PJ03 (top left panel), PJ06 (top right panel) and PJ05 (bottom left panel). For each of these crossings, the corresponding UVS maps are shown at the bottom right of the figure.

**Figure 4:**
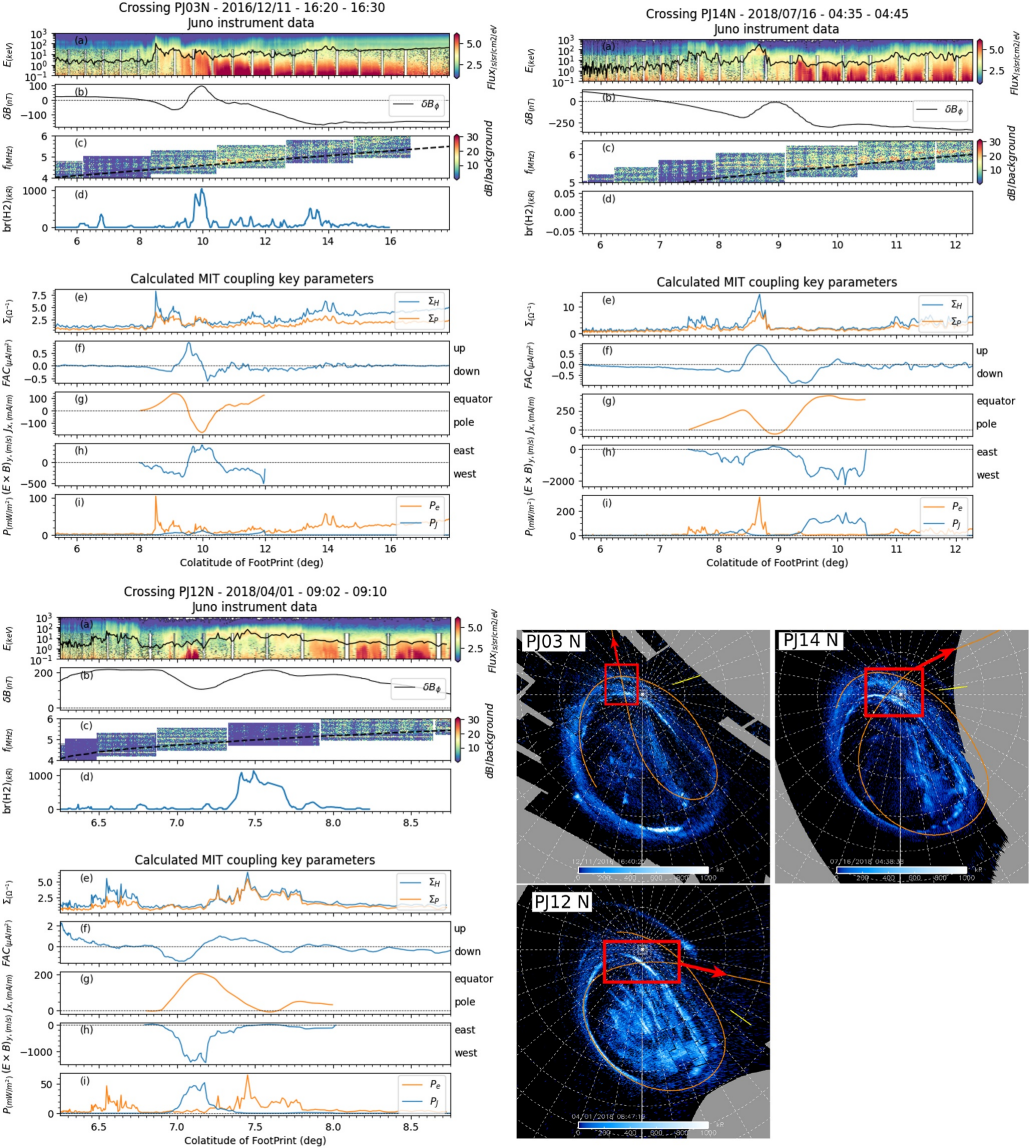
Same as Figure 3, but for northern perijoves PJ03 (top left), PJ14 (top right) and PJ12 (bottom).

We first present an overview of the auroras imaged by Juno's UVS instrument during the first 30 perijoves. The Jovian UV aurora appears very dynamic in UVS images. These UVS images reveal also a strong North/South contrast and temporal variability. For each perijove, the main oval is clearly visible (see auroral imaging in Figures 3 and 4), but with different morphologies. In both hemispheres, for some perijoves, the main oval displays a regular and smooth longitudinal and latitudinal distribution, while for others it shows strong spatial inhomogeneities at the local scale corresponding to small sectors of large brightness being adjacent to regions of much lower brightness, in a disordered manner. This complex and highly variable morphology of Jupiter's aurora have been described and classified by Grodent et al. (2018), who identified six auroral morphological families representative of the observations for the northern aurora.

The configuration of the trajectory of Juno's footprint with respect to the main aurora changes greatly from one perijove to the next one. For some perijoves, this trajectory evolves above the main aurora for significant amounts of time, while in other cases the trajectory crosses the aurora perpendicularly in a short amount of time. For this study, we consider only cases where the trajectory is perpendicular to the aurora, which is a necessary condition for the equations described by the eletrodynamics model used in this study to hold. Another necessary condition is that the local variations of the morphology of the aurora have to be much smaller along the main oval than perpendicular to the main oval, as mentioned in the description of the electrodynamics model. These two criteria were used to select the crossings for study with our method via a visual inspection of UVS images. Application of these two criteria limits the number of crossings that can be used for this study. Nevertheless, we managed to identify for this study a set of 27 crossings (13 in the northern hemisphere, 14 in the southern one) selected from the 30 first orbits of Juno. We will first present the results of the analysis done for 6 of them, namely PJ03 N, PJ03 S, PJ05 S, PJ06 S, PJ12 N and PJ14 N (where “PJXX” stands for “Perijove number XX,” and the last letter stands for North/South crossing). The auroral configurations related to these 6 crossings are displayed in Figures 3 and 4, along with the results of the study of these crossings, which have been selected for the regularity of the auroral configuration. We can also observe from the auroral image for PJ05 S that Juno's magnetic footprint crosses Io's auroral tail. These Io‐related crossings appear quite clearly in our results, as will be described later, and offer an interesting application of our method to the study of MIT coupling between Jupiter and its main satellites.

One small issue encountered in this study was the observation of a slight and consistent delay in time between the time series of UV brightness profiles at Juno's magnetic footprint measured by the UVS instrument and the data measured by the other instruments. This delay is thought to come from the uncertainties in determining the positions of the footprint of Juno from Juno's position using the magnetic field models. The choice we made in this study to address this problem has been to shift the time series of UVS brightness at Juno's magnetic footprint so as to maximize its correlation with the heating rate due to electron precipitation deduced from JADE and JEDI data. Indeed these two parameters are closely related to each other, hence the choice of maximizing the correlation between them. The corresponding time delay is given in Table 1 of the Supplementary Information for each of the six crossings mentioned above, studied in Section 3.2, for which UVS brightness time series were available. We discuss this processing in more detail in section B of the Supplementary information. We notice that this measured delay of about 1 min does not have significant consequences on the results, as the calculations in this study are mainly done using the data measured at Juno's position, independently of the position of its footprint, so that the uncertainty relative to the determination of Juno's footprint trajectory does not impact our results.

**Table 1:** Peak Values and Ranges of the Peak Values (Over All Crossings) of the Magnetosphere‐Ionosphere‐Thermosphere Coupling Parameters Calculated for Southern and Northern Crossings of the Main Auroras

	mean	range	mean	range	mean	range
	South ‐ Trend A		North ‐ Trend A		North ‐ Trend B	
Σ_*H*_ (Ω^−1^)	6	$\left[4,20\right]$	3	$\left[1,30\right]$	7	$\left[5.5,15\right]$
Σ_*P*_ (Ω^−1^)	3	$\left[1,12\right]$	2	$\left[0.8,11\right]$	4	$\left[3,8.5\right]$
FAC up (μA/m^2^)	1	$\left[0.3,2.5\right]$	0.7	$\left[0.4,1.1\right]$	0.6	$\left[0.2,0.9\right]$
FAC down (μA/m^2^)	−0.5	$\left[-2,-0.1\right]$	−0.6	$\left[-1.5,-0.1\right]$	−0.5	$\left[-0.9,-0.2\right]$
$J_{x}\;\left(\text{mA/m}\right)$	500	$\left[200,1200\right]$	300	$\left[80,700\right]$	−150	$\left[-200,-50\right]$
$J_{\text{tot}}\;\left(\text{MA}\right)$	58	$\left[23,140\right]$	35	$\left[9,81\right]$	−17	$\left[-23,-6\right]$
E × B${|}_{y}\;\left(\text{km/s}\right)$	−4	$\left[-8,-0.5\right]$	−1	$\left[-3.5,-0.2\right]$	0.65	$\left[0.15,1.4\right]$
*P*_*e*_ (mW/m^2^)	200	$\left[50,1000\right]$	80	$\left[50,400\right]$	170	$\left[100,320\right]$
*P*_*J*_ (mW/m^2^)	200	$\left[50,1000\right]$	100	$\left[50,250\right]$	90	$\left[10,180\right]$

*Note.* The results for the northern hemisphere are presented in two separate columns, associated with the two trends identified in Section 3.4.1. For easier comparison with axisymmetric models of corotation enforcement currents, an additional line below *J*_*x*_ shows the value of the total ionospheric meridional current *J*_tot_ closing the field‐aligned currents, assuming azimuthal symmetry over all local times.

### 3.2 Analysis of the Selected Cases

To validate the new version of the method which is now generalized to all perijoves, we first present two case studies that have been published in Wang et al. (2021), namely perijoves 3 and 6 southern passes. We then present a third southern perijove example followed by three examples of northern perijoves. The geometrical frame conventions used throughout this study are redefined and recalled in Figure 1. The results of the analysis of the southern passes of perijoves 3 and 6 displayed in Figure 3 top left and top right make use of these frame definitions.

In Figures 3 and 4, each sub‐figure corresponds to one of the six case studies which will be discussed. Each sub‐figure displays Juno data (four top rows) and the calculated MIT coupling key parameters (five bottom rows) as a function of the Juno magnetic footprint colatitude. The Juno magnetic footprint is computed using the JRM09 magnetic field model from Connerney et al. (2018) and the CAN81 current sheet model from Connerney et al. (1981). In each sub‐figure, the four top panels display (a) combined JADE and JEDI flux energy spectra for downward precipitating electrons (with the total energy flux, in mW/m^2^ displayed as the black curve), (b) the residual magnetic field *δ*B_*ϕ*_ perturbations (determined from MAG and JRM09 + CAN81), (c) the radio observation measured by WAVES and (d) the UV brightness profile at the Juno footprint (measured by UVS, whenever available). The five bottom panels show the key MIT coupling parameters with panel (e) the Hall (in blue) and Pedersen (in orange) conductances, panel (f) the field‐aligned currents (FAC), panel (g) the ionospheric height‐integrated *J*_x_ current (perpendicular to the main oval), panel (h) the azimuthal component of the west/east E × B|_y_ drift in the upper atmosphere and finally panel (a) the particle (orange) and Joule (blue) heating rates. The polar projection maps reconstructed from UVS data for each of these selected perijoves are displayed in the bottom right‐hand of the figures, with the main aurora crossing highlighted by the red box.

As described briefly in Section 2.2 and also described more precisely in section D of the Supplementary information, the relative uncertainties over the MIT coupling key parameters plotted in panels (g) to (i) are sensitive to the width of the interval on which these parameters are calculated. For this reason, the data in panels (g) to (i) are only plotted for selected portions of Juno's trajectory for which the error bars are well controlled.

In each of these crossings, the main oval regions can easily be identified in the multi‐panels plots by the enhancement in the UV brightness profile (panels (d), whenever available) and the enhanced downward electron total energy flux (panels (a), solid black curve).

We first focus on the crossing of the southern auroral oval at perijove 3 (Figure 3 top left) and perijove 6 (Figure 3 top right), which we compare to the results of Wang et al. (2021, see its Figures 7 and 8). Despite a different determination of the *δB* components perturbation (see Section 2.2), we obtain the same results for the perijove 3 crossing (see Figure 3 top left), namely upward FAC on the equatorial edge of the main oval adjacent to downward FAC poleward of it. During these FAC crossings, we measure a strong increase of both the Hall and Pedersen currents, an equatorward *J*_x_ current, a westward azimuthal component of E × B|_y_, and a simultaneous increase of particle and Joule heating rates.

In perijove 6 southern crossing (see Figure 3 top right), the same behavior is observed for the electron precipitation spectra (panel (a)), for the resdiual magnetic field B_ϕ_ (panel (b)), for the Hall and Pedersen conductances (panel (e)), and for the field‐aligned currents (panel (f)). Looking at the UVS data (panel (d), and related UVS imagery in the figure) we can see that Juno crosses two main structures. It crosses first the main oval, corresponding to the higher increase in brightness at ∼163.9°‐164.3° of colatitude, and identified in the plots with a blue background. Then, at higher colatitude, it crosses a second structure corresponding to a less intense brightness from the UVS data but with a reasonable enhancement in the total energy flux within the loss cone (black curve in panel (a)), and identified in the plots with an orange background. This suggests that two distinct structures have been crossed successively.

The first main structure (corresponding to the main oval) displays a strong upward current whose peak is reached at ∼164.3° of colatitude. Two weak downward currents of similar amplitude can be identified on the equator side and on the polar side of this large upward current. This configuration is associated with poleward ionospheric *J*_x_ current at lower colatitudes (∼163.5°‐164.3°), and to equatorward ionospheric *J*_x_ current at higher colatitude (∼164.3°‐165°), closing in the ionosphere the three layers of field‐aligned currents associated with this first structure. The second structure displays only a downward current associated with a poleward ionospheric *J*_*x*_ current, identified with the orange background in the figure. These two different regions are in good correspondence with “Region I” and “Region II” identified by Mauk et al. (2020) (see their figure 13 and the definition of these two regions in their study), with mostly downward electron acceleration in “Region I,” while the second structure displays downward proton inverted V embedded inside a “Region II.” The secondary structure likely represents a particular case of a downward field‐aligned current sheet not directly related with the main oval observed equatorward of it.

The identification of these two main structures is consistent with the analysis of Kotsiaros et al. (2019) and Wang et al. (2021). The slight nuance brought by our new method consists in identifying downward field‐aligned currents attached to both sides of the first structure, which leads us to suggest that the two structures are in fact independent. This is supported by the observation of a central region of zero field‐aligned current between the two structures, and from the UVS imagery and data.

We then analyze a third crossing of the southern auroral oval during perijove 5 (Figure 3 bottom). An increase in both the total downward electron energy flux (panel (a)) and UV brightness profile (panel (d)) is observed between ∼165.7° and ∼168.9°. The southern crossing from perijove 5 is distinct from the ones from perijove 3 and 6 in the sense that the calculated field‐aligned currents display quickly evolving variations in regions at lower colatitude than the main oval, between ∼160° and ∼163.5°. These variations are of amplitude comparable to the amplitude of the field‐aligned currents associated with the crossing of the main oval. This shows that either the FAC associated with the crossing of the main oval are of weak amplitude, or the FAC associated with other phenomena not taken into account display a dominant contribution. In any case, from the results, we observe for this crossing a layer of upward FAC, followed at lower colatitude by a layer of downward FAC. These two layers are separated by a region of zero FAC. An equatorward ionospheric current and a westward E × B|_*y*_ azimuthal drift are associated with this crossing.

We consider now the study of the northern auroral oval crossings. The first crossing we consider is PJ03 N, associated with the perijove 3. Figure 4 (top left panels (a) to (d)) display the Juno measurements during the crossing, while Figure 4 (top left panels (e) to (i)) display the calculated MIT key parameters. An enhancement is observed in downward electron energy flux (though weaker than for the southern passes) from ∼8.1° to ∼9° of colatitude. The calculated MIT key parameters show enhancements in the Hall and Pedersen conductances as well as in electron precipitation heating rate. Upward FAC are observed in the polar side of the main oval, and downward FAC are observed in the equator side. Ionospheric *J*_*x*_ currents flow equatorward on the polar edge of the crossing as well as on the equator edge, and poleward in the central region where the field‐aligned currents revert sign. E × B|_y_ drift are westward on the two edges of the crossing and eastward in the central region.

Figure 4 (top right) displays the Juno data and MIT coupling key parameters for perijove 14. This crossing of the main oval is very similar to perijove 3 (Figure 4 top left). A peak is observed in both the Hall and Pedersen conductances, as well as in electron precipitation heating rate. An upward field‐aligned current is observed in the polar side of the main oval, and a downward field‐aligned current is observed in the equator side. A poleward ionospheric *J*_*x*_ current is observed in the central region where the field‐aligned currents reverse direction, surrounded by equatorward ionospheric *J*_*x*_ current on both sides. E × B|_y_ drifts are eastward in the central region between the two layers of fields aligned currents, and westward on both sides of the main oval.

Finally, Figure 4 (bottom) displays the main oval crossing that occurs during perijove 12. This crossing shows a opposite configuration to the ones discussed with perijoves 3 and 14. During this crossing, we observe a downward field‐aligned current in the polar side of the main oval, and an upward field‐aligned current on the equator side. The ionospheric *J*_*x*_ current is mostly equatorward, and the E × B|_y_ ionospheric plasma drift is mostly westward in the central region and in both sides. The peaks of the particle heating rate and Hall and Pedersen conductances are observed in the polar side of the FAC system.

For almost all northern and southern hemisphere passes, a radio emission source is observed (panels (c)) at a frequency close to the local electron cyclotron frequency (dashed black line) equatorward of the main oval crossings, each time at a few degrees of latitude from the UV brightness peak.

These first case studies already allow us to identify some trends. In each southern crossing case, there is a peak in the Hall and Pedersen conductances (Figure 3 panels (e)) and Joule and particle heating rates (Figure 3 panels (i)) above the main oval. All current systems display upward FAC on the equator side, and downward FAC on the polar side (Figure 3 panels (f)). This structure is associated with an equatorward ionospheric current *J*_x_ (Figure 3 panels (g)) and a mostly westward E × B|_y_ drift (Figure 3 panels (h)).

In contrast, one does not find such a clear trend in the northern hemisphere. In each of the three cases, there is a peak in Hall and Pedersen conductances (Figure 4 panels (e)) and in electron precipitation heating rate, but not always in Joule heating rate (Figure 4 panels (i)). Part of the current systems (perijoves 3 and 14) display an upward current in the polar side, and a downward current in the equator side, with peak values almost half those of the southern hemisphere. However, perijove 12 displays an opposite configuration, with a downward FAC in the polar side and upward FAC in the equator side. Ionospheric currents *J*_*x*_ and E×B|_y_ drifts display different behaviors: for crossing PJ03 N (Figure 4 top left), and PJ14 N (Figure 4 top right) a central region of poleward ionospheric currents is surrounded by regions of equatorward ionospheric currents, and a central region of eastward E × B|_y_ drifts is surrounded by regions of westward drifts. But, for PJ12 N (Figure 4 bottom) ionospheric currents flow in only the equatorward direction, and E × B|_y_ drifts only in the westward direction, as observed in the southern hemisphere.

### 3.3 Case Analysis of Io Flux Tube Crossings

**Figure 5:**
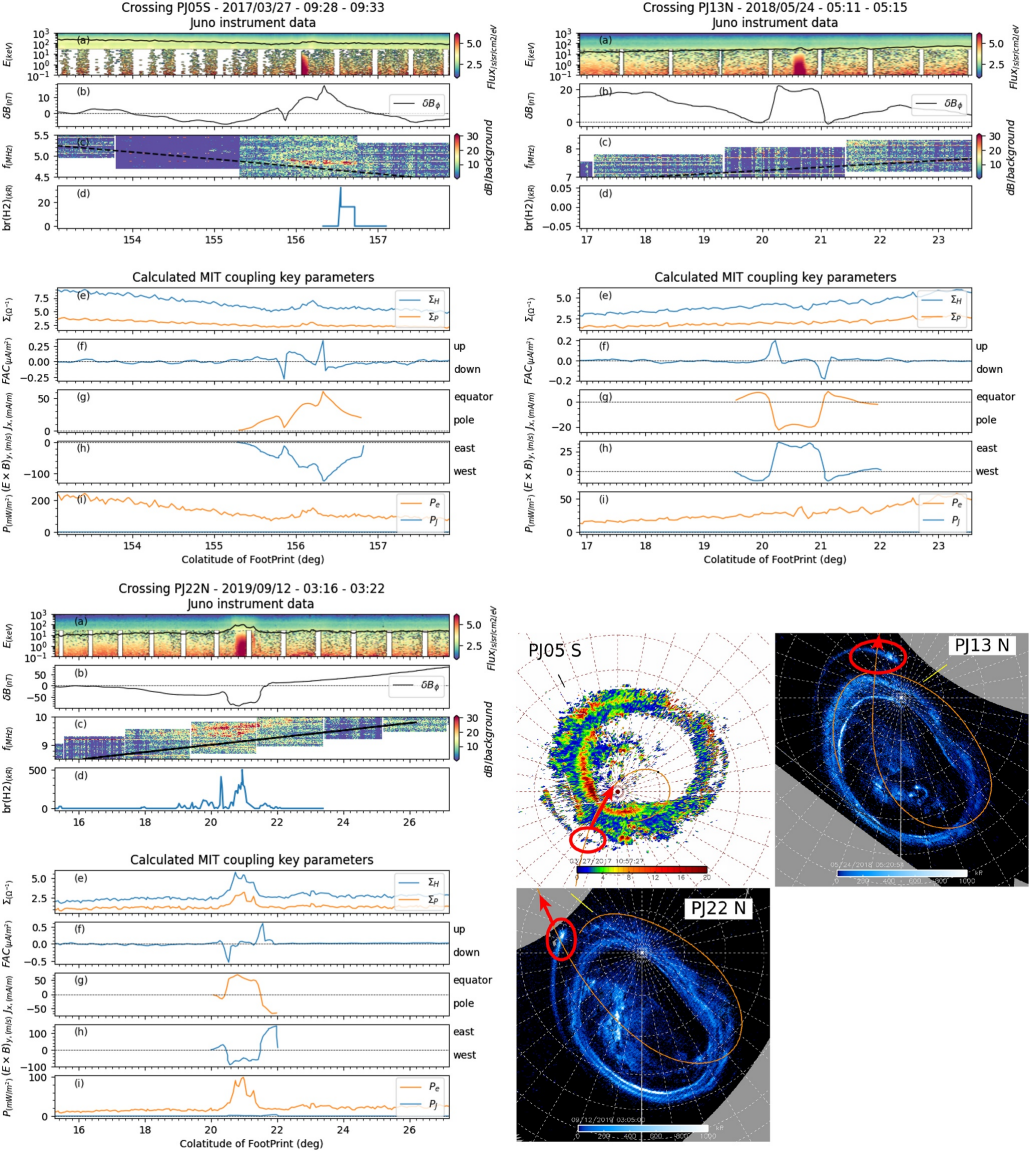
Same as Figure 3, but for Io's tail flux tube crossings. UVS imagery for perijove five is shown in units of color ratio rather than in kR to enhance the visibility of the crossing between the trajectory of Juno's footprint and Io's tail.

Unexpectedly, it turns out that the method used to analyze the key parameters of MIT coupling above the main oval also allows us to study the crossing of the flux tubes connected to Io's auroral tail. Figure 5 displays three examples. In these regions (already studied by Szalay et al., 2018, 2020), we observe a peak in the downward flux of electrons measured by JADE/JEDI (panel (a)), as well as a perturbation of the *δB*_*ϕ*_ component (panel (b)).

In the case of the southern hemisphere crossing (PJ05, Figure 5 top‐left), application of our method reveals the presence of a small increase in the Hall and Pedersen currents (panel (e)), and in particle heating rate (panel (i)). The absence of effect of these concurrent variations on Joule heating rate variations can be explained by the fact that Joule heating increases with increasing current intensity and decreases with increasing Pedersen conductance (due to particle precipitation increase), thus the two effects compensate each other. A downward FAC is observed on the equator side of the crossing, and an upward FAC is observed of the polar side, separated by an area of near‐zero FAC (panel (f)). Between these FAC, an equatorward *J*_*x*_ ionospheric current and a westward E × B|_y_ drift are observed, where clear Alfvénic fluctuations were measured by Gershman et al. (2019) and Szalay et al. (2018). During this Io's flux tube crossing, the source of a DAM radio emission induced by Io is crossed (Louis et al., 2019), as can be observed from WAVES data (panel (d)).

The top‐right panels of Figure 5 display a northern Io tail crossing during perijove 13. In this case, we do not observe any significant increase in the Hall and Pedersen conductances (panel (e)) or in the Joule heating rate (panel (i)). A very small increase is observed in the particle heating rate (panel (i)). A downward FAC is observed on the equator side of the crossing, and an upward FAC is observed on the polar side, separated by an area of near‐zero FAC (panel (f)). In this case, a poleward *J*_*x*_ ionospheric current and an eastward E × B|_y_ drift are observed. No radio emission is observed during this crossing.

Finally, the bottom panels of Figure 5 display a second northern Io tail flux tube crossing during perijove 22. This case shows a larger increase in both the Hall and Pedersen conductances (panel (e)) and in electron precipitation heating rates (panel (i)). The observed configuration of FAC in this case is opposite to the first norhern Io trail crossing described above. An upward FAC is observed in the equator side, and a downward one is observed in the polar side, separated by an area of near‐zero FAC. This configuration is associated with equatorward *J*_*x*_ ionospheric currents and eastward E × B|_y_ drifts.

In two cases (top‐left and bottom‐left panels) an increase in the Pedersen and Hall conductances and particles heating rate is observed, associated with an equatorward *J*_*x*_ ionospheric current and a westward E × B|_y_ drift, but with opposite FAC system. A radio emission (panels (c)) is observed very close to the electron cyclotron frequency (dashed black line), indicating that Juno flew very close to (if not inside) the radio source. In the third case (top‐right panel), the FAC are associated with a poleward _x_ ionospheric current and an eastward E × B|_y_ drift, while no significant conductances are observed. In that case, no DAM radio emission is observed during the flux tube crossing.

### 3.4 Statistical Analysis

#### 3.4.1 Superposed Analysis

Having analyzed in the previous section three case studies per hemisphere, we now turn to the statistical study of the crossings of the magnetic field lines connected to the main oval for several of the first 30 perijoves.

**Figure 6:**
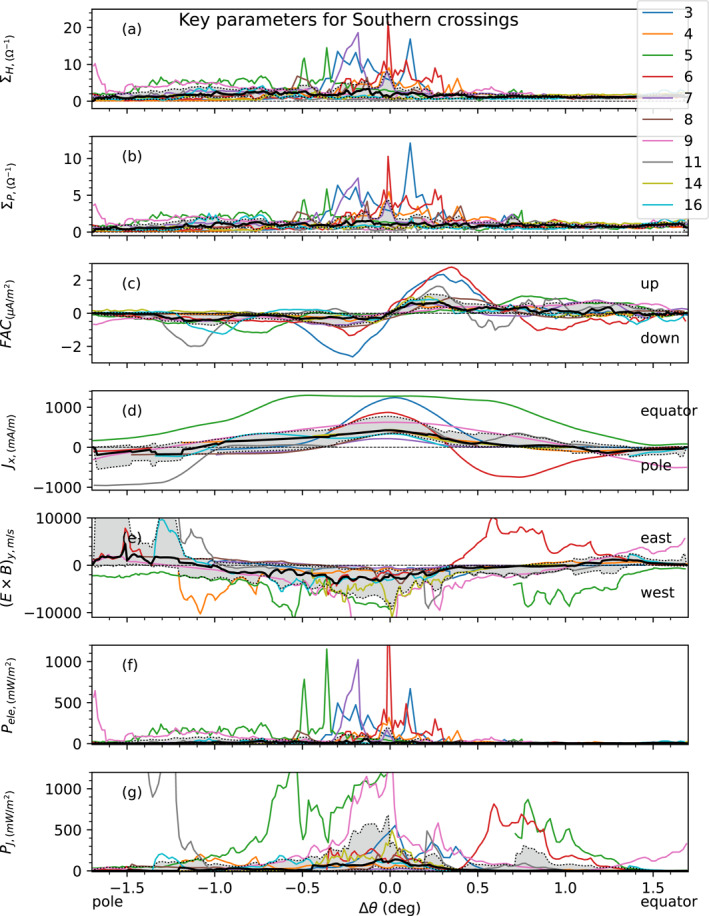
Superposed analysis of key Magnetosphere‐Ionosphere‐Thermosphere coupling parameters calculated above the southern Main Oval as functions of Δ*θ* = *θ*_Footprint_ − *θ*_ref_, where *θ*_Footprint_ is the colatitude of the footprint of Juno and *θ*_ref_ is the colatitude of reference, defined at the colatitude where Field‐aligned Currents change direction. Curves with different colors correspond to the different perijoves. For a given value of Δ*θ*, the black curve corresponds to the median taken over the crossings, and the shaded area corresponds to value between the 0.2 and the 0.8 quantiles over the crossings. (a): calculated height‐integrated Hall conductance, (b): calculated height‐integrated Pedersen conductance, (c): field‐aligned currents calculated with our electrodynamics model, (d): ionospheric height‐integrated currents for the x component, the perpendicular direction to the main oval, (e): azimuthal component of ionospheric E × B drift, (f): electron precipitation rate per unit column of atmosphere, (g): Joule heating rate per unit column of atmosphere.

**Figure 7:**
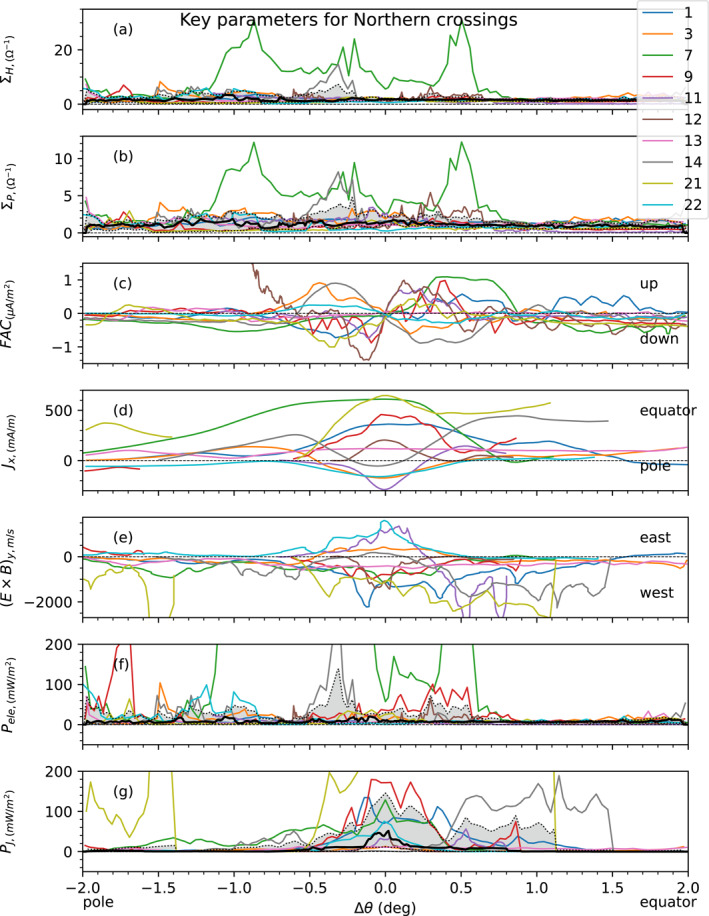
Same as Figure 6 for the northern main oval, for a selected set of perijoves. Analysis of panel (c) shows that there are at least two trends of opposite behavior. Trend A (PJ 1, 7, 9, 11, 12, 13, 21) corresponds to the occurrence of a downward field‐aligned current in the polar side of the central point, with an upward field‐aligned current in the equator side. Trend B (PJ 3, 14, 22) corresponds to the opposite trend, with an upward field‐aligned current in the polar side, and a downward one in the equator side. The statistical dispersion for the parameters plotted in panels (c) to (e) are not plotted, as the average is taken over two trends of opposite contributions, thus averaging trivially to zero. Instead, average values and dispersions for both trends are described in Table 1.

One major feature that was common to almost all the crossings considered in this study was the observation of a well‐defined point along Juno's magnetic footprint at which the FAC change direction. This feature was clearly apparent in all the cases previously presented. We use this repeatable feature to define, for each crossing, the parameter *θ*_inv_ which corresponds to the colatitude at which we observe this inversion in the direction of FAC. This parameter serves a reference point to perform a superposed analysis of the main aurora crossings from all north perijoves, and likewise for all south perijoves. Our aim is to study the profile of latitude variations of the key parameters with respect to this arbitrary point. The results of this superposed analysis for each key MIT parameter are presented in Figures 6 and 7, for the southern and northern hemispheres, respectively. The different perijoves studied are distinguished via color coded curves indicated on the right‐hand side of the figures. The black curve represents the median of all curves and the gray shaded area the region between the 0.2 and the 0.8 quantiles. These results are presented as a function of Δ*θ* = *θ*_Footprint_ − *θ*_inv_, the distance in colatitude to the arbitrary defined point *θ*_inv_, with the polar side region on the left‐hand side and the equator side region on the right‐hand side of the figures. One can see from panel (c) that Δ*θ* = 0 was chosen as the location where FAC change direction (Figures 6c and 7c).

We focus in this paragraph on Figure 6 presenting the results of this analysis for the southern hemisphere. Panel (c) shows that there is a strong tendency for upward field‐aligned currents on the equator side of the reference point, and a downward field‐aligned currents on its polar side. Then panel (d) shows that these FAC close in the ionosphere via an equatorward ionospheric current *J*_*x*_. The E × B|_y_ drift are westward close to Δ*θ* = 0, indicating the presence of a region of sub‐corotation. These observations are statistically significant, since the shaded areas share the same features. Enhancement in both Hall and Pedersen currents, as well as in Joule heating rates, are also observed in the central region. On each side of this central region, where one could expect ionospheric currents flowing in the opposite direction, no significant trend emerges. This probably is a consequence of the large uncertainty of this analysis in the regions of small conductances.

We now focus on the northern hemisphere in Figure 7. Inspection of the results shows at first that the trends are not so clear as they were for the southern hemisphere. One can first observe from panels (a) and (b) a consistent increase in Hall and Pedersen conductances poleward of the central region. Then panel (c) displays upward and downward currents on both sides of the central region, without a clearly dominant trend. Ionospheric currents (panel (d)) are distributed on the two sides of the *J*_*x*_ = 0 curve in the central region. Similarly, the distribution of the drift velocity E × B|_y_ does not show an overall clear tendency over all perijoves: some perijoves indicate super‐corotation of the azimuthal ionospheric flow plasma, while others show sub‐corotation. However, further inspection of panel (c) shows that at least two opposite trends can be identified, which are of opposite behavior. Trend A (associated with PJ 1, 7, 9, 11, 12, 13, 21) corresponds to the occurrence of a downward field‐aligned current in the polar side of the central point, with an upward field‐aligned current in the equator side, similar to what was observed in the southern hemisphere. Trend B (associated with PJ 3, 14, 22) corresponds to the opposite trend, with an upward field‐aligned current in the polar side, and a downward one in the equator side. In that regard, the statistical dispersion of field‐aligned current (panel (c)), the ionospheric current (panel (d)) and the azimuthal ionospheric drift velocity (panel (e)) were not considered over the entire set of crossings, as the average would have been taken over two trends of opposite contributions, thus averaging to zero. Instead, the statistical dispersions were considered individually for each of trend A and B. The results are described in Table 1, and will be discussed further below. The remaining crossings (PJ 4, 16, 20) correspond to cases where no dominant trend have been observed despite the presence of peaks of ionospheric conductances and strong signatures of FAC in the residual magnetic field. Indeed, in theses cases, we were not able to identify a precise point *θ*_inv_ at which we observe an inversion in the direction of FAC, so these cases were not shown in Figure 7, but there implications on our understanding of MIT coupling at Jupiter are discussed in the next section.

#### 3.4.2 Conductances and Heating Rates

**Figure 8:**
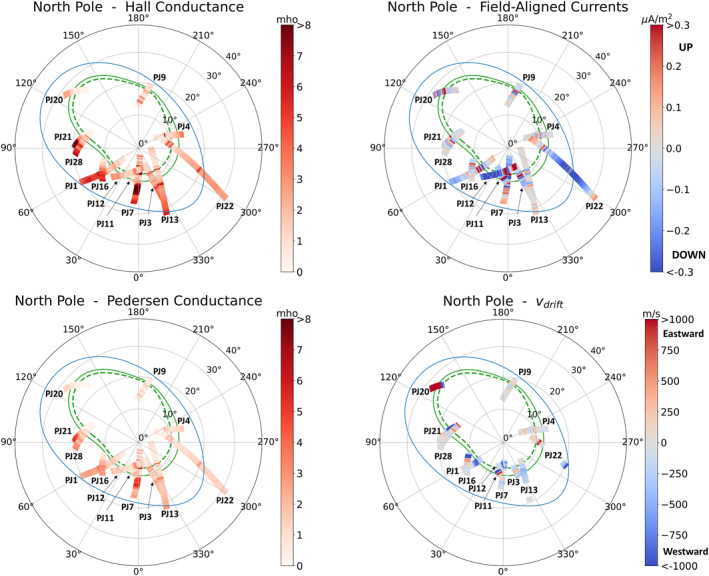
Superposed analysis of the variations of Magnetosphere‐Ionosphere‐Thermosphere coupling parameters across the southern main aurora, in planetocentric coordinates (latitude and longitude). The statistical main oval is plotted in green (inner oval is dotted and outer oval is solid) and the Io magnetic fooprint is plotted in blue. Top left panel: calculated ionospheric Hall conductance. Top right panel: calculated ionospheric pedersen conductance. Bottom left panel: calculated field‐aligned currents. Bottom right panel: ionospheric E × B drift velocity, where red colors correspond to eastward velocities, and blue colors correspond to westward velocities.

**Figure 9:**
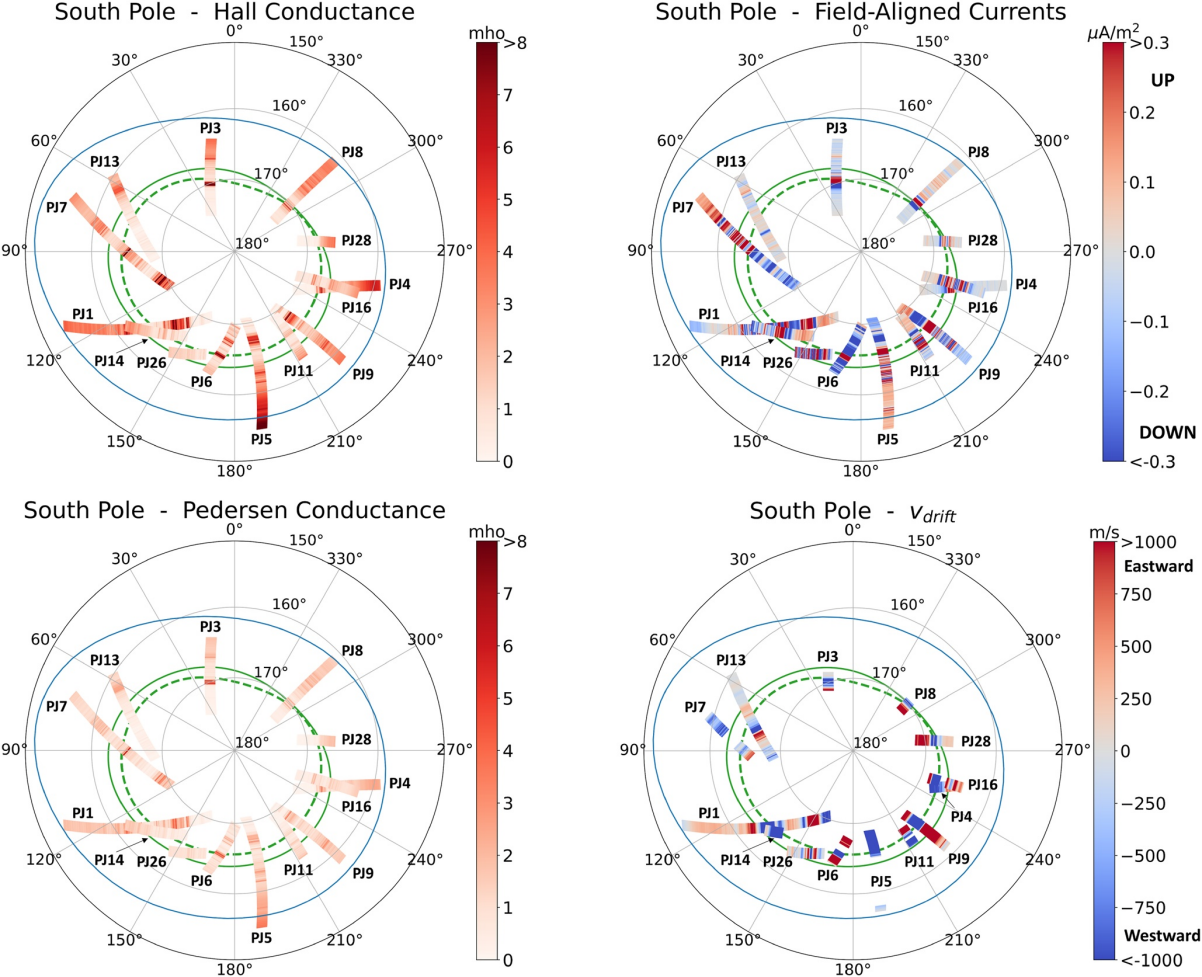
Same as Figure 8 for the northern aurora.

To study how MIT coupling parameters vary with longitude along the main oval, we also plot the MIT coupling key parameters in planetocentric coordinates in Figures 8 and 9. The statistical main oval, which does not necessarily correspond to the location of the oval prevailing at the time of each crossing, is also plotted. Hall as well as Pedersen conductances (top panels) are seen to increase near the oval. FAC (bottom left‐hand panel) as well as drift velocities (bottom right‐hand panel) are quasi null except in the regions near the oval. Drift velocities oscillate between negative and positive values, that is, between super‐corotation and sub‐corotation. Careful inspection of the south pole figure reveals that the drift velocity is negative in the inner oval and positive in the outer oval: a “blue” (i.e., negative) region is surrounded by two “red” (i.e., positive) regions.

## 4 Discussion

### 4.1 Summary of the Main Trends

Considering all crossings selected for this study, we can now summarize the main trends in our calculated MIT coupling parameters. The main excursion ranges and typical peak values of these parameters calculated for the north and south main aurora crossings are described in Table 1.

For an extensive discussion of the computation of the relative uncertainties over the calculated MIT coupling key parameters, we refer the reader to section D of the Supplementary Information document. We summarize here for the sake of completeness the main conclusions. We estimate to 20% the relative uncertainties over the ionospheric Hall and Pedersen conductances *Σ*_*H*,*P*_ and over the particle heating rate *P*_*e*_, with respect to the values displayed in Table 1. This means that we have ΔΣ_*P*_/*Σ*_*P*_ = ΔΣ_*H*_/*Σ*_*H*_ = Δ*P*_*e*_/*P*_*e*_ = 0.2. We also estimate to 20% the relative uncertainties over *J*_‖_ and *J*_*x*_, respectively the field‐aligned current and the ionospheric current perpendicular to the main oval. This means that we have Δ*J*_‖_/*J*_‖_ = 0.2, and also Δ*J*_*x*_/*J*_*x*_ ≤ 0.2 wherever *J*_*x*_ is calculated. The motivation behind computing only *J*_*x*_ on a few selected intervals of colatitude are described more extensively in the Supplementary Information document, section D. Finally, we have Δ*P*_*J*_/*P*_*J*_ = *Δ*(E × B)/(E × B) ≤ 0.4, the relative uncertainties over the azimuthal drift velocity E × B and the Joule heating rate *P*_*J*_.

With due consideration of these uncertainties, some common characteristic behaviors of the variations of the MIT coupling key parameters across the main aurora appear to apply to all perijove crossings. Almost systematically, one can identify a main time interval during which particle precipitation rates and ionospheric Hall and Pedersen conductances simultaneously reach their largest values. This period generally coincides with the presence of a pair of adjacent upward and downward field‐aligned current flows within which these FAC reach their largest intensities. This pair of FAC closes horizontally through the ionospheric conductor via meridional ionospheric Pedersen currents. These ionospheric currents flows in the equatorward direction when the upward FAC is on the equator side of the downward FAC. Conversely, these ionospheric currents flow in the poleward direction when the upward FAC is located on the polar side of the downward FAC. As a direct consequence of our use of the ionospheric Ohm's law in our calculations, poleward meridional currents are associated with poleward ionospheric electric fields and eastward E × B plasma drifts corresponding to super‐corotation. Conversely, equatorward meridional currents are associated with equatorward electric fields and westward E × B drifts corresponding to sub‐corotation.

**Figure 10:**
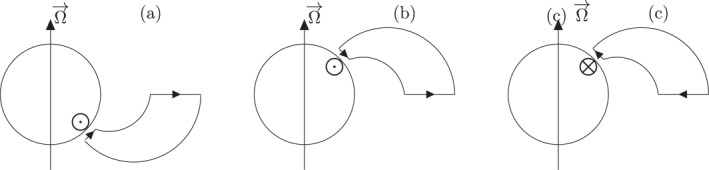
Cartoon showing the two different trends in Magnetosphere‐Ionosphere‐Thermosphere coupling of the main aurora region to the magnetodisk emerging from this study. In trend A, represented in panel (a) for the southern hemisphere (corresponding to all southern crossings studied) and in panel (b) for the northern hemisphere (corresponding to PJ 1, 7, 9, 11, 12, 13, 21), the ionosphere‐magnetosphere current loop transfers angular momentum from the planet to the magnetodisk, enforcing its corotation and accordingly dragging the ionosphere into sub‐corotation. In trend B, observed for several of the northern perijoves (corresponding to PJ 3, 14, 22) as illustrated in panel (c), angular momentum is transferred from the magnetodisk to the planet and accelerates the ionospheric plasma into super‐corotation.

This analysis allows us to identify clear trends emerging for the latitudinal variations of our calculated MIT coupling parameters across the main aurora, which are present in totally different proportions in the two hemispheres. Figure 10 illustrates how these trends can be translated into two oppositely directed current loops connecting the ionosphere to the magnetodisk. The first trend, which we call trend A corresponding to about half of the studied cases (7/13), is associated with a system of equatorward ionospheric currents connecting a downward FAC on the polar side to a an upward FAC on the equator side. In this configuration, the ionosphere‐magnetosphere current loop has to close via a radial outward current in the magnetodisk. In this current loop, the corresponding ExB Lorentz force accelerates the magnetodisk in the direction of corotation, and accordingly decelerates the ionospheric E × B plasma, producing sub‐corotation in the ionosphere. Angular momentum is transferred from the planet to the magnetodisk to partially enforce its corotation, in line with the models of Hill (2001) and Cowley and Bunce (2001). This trend fully prevails in all studied southern perijoves (see Figure 10a). It is also observed over the major part of the northern perijove crossings considered in this study (7 crossings out of the 13 studied in the north), as represented in Figure 10b. Taking into account the peak mean value of the ionospheric current mentioned in Table 1, we estimate the total ionospheric current flowing toward the equator to be about 9.3 MA/rad in the southern hemisphere and about 5.6 MA/rad in the longitudinal sectors in the northern hemisphere where trend A was observed. This estimation is expressed in units of total current per radian of azimuth.

Conversely, the second trend, which we call trend B and corresponding to about a quarter of the studied cases (3/13), is associated with poleward ionospheric currents connecting a downward FAC on the equator side to an upward FAC on the polar side. In this configuration, the ionosphere‐magnetosphere current loop has to close via a radial inward current in the magnetodisk, which instead accelerates the ionospheric E × B drift into super‐corotation and decelerates the magnetodisk into sub‐corotation. This transfers angular momentum from the disk to the planet, contrary to the corotation enforcement model. This second trend, illustrated in Figure 10c, is observed over 3 of the 13 north perijove fly bys, corresponding to PJ 3, 14 and 22 N. Taking into account the peak mean value of the ionospheric current mentioned in Table 1, we estimated the total ionospheric current flowing toward the pole to be about 2.8 MA/rad in the longitudinal sectors where trend B was observed. The remaining cases with no associated trend, corresponding to about a quarter of the studied cases (3/13), reflect coupling processes of higher complexity than what can be described by the overly simplified trends described in Figure 10.

The magnitude of currents in the current loops we find here connecting the ionosphere and magnetosphere can be compared with the predictions of Cowley et al. (2008) and Cowley et al. (2017) made in anticipation of Juno observations (see Figure 1 of Cowley et al., 2008). The latitudinal extension of these closing currents is on the order of 1.5° to 2° in our study, with about 0.7° to 1° of extension for both upward and downward field‐aligned currents, as can be seen in our Figures 6 and 7. This can be compared to a significantly broader current closure region predicted by Cowley et al. (2008): on the order of 2° for upward currents on the equatorward side, and of 4° to 5° for the downward currents on the poleward side (see their panel c). Their prediction of a total meridional current intensity on the order of 20–40 MA (their panel b) is very similar to the values deduced from our observations and shown in Table 1 (60 MA for the southern hemisphere, 30 MA for the northern hemisphere in trend A). Finally, they predict upward field‐aligned current intensities on the order of 300 nA/m^2^, about a factor of 3 smaller than our estimates, and downward field‐aligned current intensities on the order of 50 nA/m^2^, about a factor of 10 smaller than ours, as already noticed by Wang et al. (2021). Our observed field‐aligned currents are actually larger than their predictions but flow over a correspondingly smaller latitude band, thus producing ionospheric closure currents of a similar magnitude as their prediction. Overall, our observations reveal significantly more narrowly confined field‐aligned currents than in the model predictions of Cowley et al. (2008) but remarkably similar total currents in the MIT coupling current loops. However, this narrowing of the region of field‐aligned currents in comparison to the predictions of the “standard” Cowley‐Bunce model has been proposed by Nichols and Cowley (2004) and later by Ray et al. (2015), to be an effect of the amplification of the Pedersen conductance by enhanced electron precipitation associated with these upward field‐aligned currents. This lower value of the latitudinal width of upward field‐aligned currents associated with the main ovals is consistent with our observations.

Joule heating rates across the main aurora display peak values of the order of one to a few hundreds of mW/m^2^, of the same order of magnitude as particle deposition rates, as can be seen from inspection of Table 1. Bougher (2005) proposed a Jupiter Thermospheric General Circulation Model in which large Joule heating rates are calculated to be 70 mW/m^2^ in the northern oval region, and nearly double (up to 140 mW/m^2^) in the southern auroral oval. Tao et al. (2009), on the other hand, proposed a model that includes the effects of neutral dynamics on the coupling current: the peak values found around latitude 75° in their model are about 100 mW/m^2^. Finally, in the model of Ray et al. (2015) the maximum auroral precipitation intensity predicted, the equivalent of our *P*_*e*_, is 60 mW/m^2^. The corresponding Joule heating rate is not directly provided in their publication but it can be deduced from their calculated field‐aligned current and ionospheric electric field latitudinal profiles assuming current continuity (see their Figure 9), leading to a Joule heating rate on the order of 120 mW/m^2^. These three different models thus predict similar Joule heating rates, with peak values on the order of 100 mW/m^2^, consistent with the values we infer from Juno observations in the present study.

In addition to main aurora crossings, we also analyzed a few Io footprint crossings, revealing a clear signature in field‐aligned current latitudinal variations with peaks of similar amplitude and opposite signs separated by a central region of negligible FAC intensities. The relative positions of the downward and upward peaks were inverted with respect to each other in the two northern hemisphere cases. The inversion of the FAC observed during these two flux tube crossings could be explained by the complex structure of the Io's tail and the reflection of the Alfvén wings on the Io's torus and/or on the ionosphere (as proposed by Jacobsen et al. (2007, 2010); Schlegel & Saur, 2022), but the study of a larger number of crossings is needed to investigate this further. No consistent signature in particle data emerged from the selected crossings, but a clear signature in particle heating rate was found in the PJ22 N case. WAVES data show that these crossings are associated in two over three cases with observation of radio emissions. Our study of Galilean moons auroral tails, though limited by the small number of crossings studied, suggests that this method could be used in the future for a more systematic study of the electrodynamic coupling between Jupiter and these moons.

### 4.2 Comparison With Previous Observations of MIT Coupling Parameters: Field‐Aligned Currents and Ionospheric Drifts

The systematic study of field‐aligned currents by Kotsiaros et al. (2019) uses the MAG instrument. Their study covered both hemispheres and reveals a significant asymmetry between them. Our calculated field‐aligned current systems display a similar configuration for the southern hemisphere, with upward field‐aligned currents on the equatorward side of downward ones. In contrast, we do not observe such a repeatable pattern for the northern hemisphere. As seen in Figure 10, two opposite configurations are observed among all fly bys: (a) upward currents on the equator side of downward ones and (b) downward currents on the equator side of upward ones.

**Figure 11:**
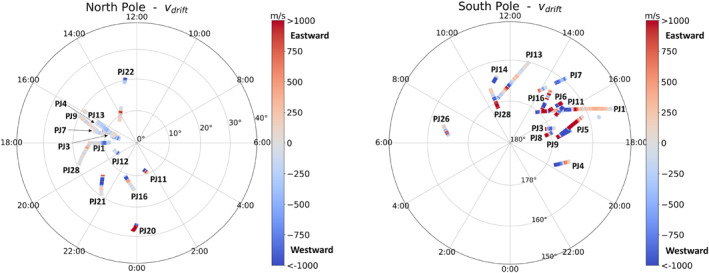
Magnetosphere‐Ionosphere‐Thermosphere distributions of E × B ionospheric drifts calculated by our method for the northern (panel (a)) and southern hemisphere (panel (b)). Red colors correspond to eastward velocities, and blue colors correspond to westward velocities.

Our calculated E × B ionospheric drifts are plotted as a function of magnetic local time (MLT) for the two hemispheres in Figure 11. Due to the slow drift of the Juno orbit with respect to the Sun‐Jupiter line, they cover only a fraction of all MLT, mainly from 16:00 to midnight for the northern hemisphere, and from 12:00 to 20:00 for the southern hemisphere. The northern hemisphere drifts can be compared with the observations of ${H_{3}}^{+}$ ion drifts by Johnson et al. (2017) using the CSHELL instrument previously available at the NASA IRTF in Hawaii (Stallard et al., 2003) and the CRIRES instrument on the VLT. In the same MLT sector, as seen in their Figures 8 and 10, Johnson et al. mainly saw weak sub‐corotational flows in the range of one km/s extending on the two sides of the main oval (or maximum of ${H_{3}}^{+}$ emission). In our study, similarly weak subcorotational flows are also consistently seen in the southern hemisphere over the region of maximum ionospheric conductances, which corresponds to the main oval and auroral emissions (Figure 6). But in the northern hemisphere, which is more directly comparable with the VLT observations, these weak corotational flows are found mainly equatorward of the region of maximum conductances, whereas weak flows in both directions are found over the broad magnetic latitude range where ionospheric conductances maximize.

Regarding our calculated field‐aligned current flows, as in Kotsiaros et al. (2019), we find that the mean total field‐aligned current is twice as large in the southern hemisphere (see Table 1) as in the northern hemisphere. Since no such significant asymmetry is found in the ionospheric conductances, consistent with Gérard et al. (2020), an explanation for this FAC inter‐hemispheric asymmetry likely resides with the inter‐hemispheric asymmetry of the distribution in longitude of the planetary magnetic field magnitude. Analysis of Figure 2 of Connerney et al. (2018) shows that the magnetic field magnitude remains almost constant along the south main oval, varying from 8 to 12 G, while it varies much more along the north main oval going from 6 to 20 G. This conclusion largely still holds when considering the latest Juno‐derived magnetic field model (JRM33 magnetic field model + CAN20 current sheet model from Connerney et al., 2020, 2022).

### 4.3 Comparison With Models of Plasma Convection in the Ionosphere and Magnetosphere

The local time coverage of E × B drifts shown in Figure 11 allows only a limited comparison with global ionospheric convection models. Taken over the two hemispheres together, sub‐corotational flows in the km/s range are dominant, consistently with the axisymmetric corotation enforcement models of Cowley and Bunce (2001) and Cowley et al. (2005). These westward flows in the afternoon sector are also consistent with the more qualitative, but local‐time dependent model of Cowley et al. (2003), in which sub‐corotation flows due to corotation breakdown co‐exist with tailward flows at higher latitudes driven by the Vasyliunas cycle in the global pattern of horizontal flows in this local time sector.

In contrast, when the same comparison is done separately for each hemisphere, a fair consistency with these model predictions is found for the southern hemisphere, where sub‐corotation seems to prevail (Figure 10a). In contrast, over the northern hemisphere, sub‐corotation flows dominate on the equator side of the main oval, whereas weak flows in the two opposite directions are found among all perijoves over the region of main emission, as illustrated in Figures 10b and 10c.

This difference in the general trend of E × B flows between the two hemispheres, with a less systematic pattern and more variability from orbit to orbit found in the northern than in the southern hemisphere, echoes the differences in FAC patterns found by Kotsiaros et al. (2019) and in this study. It suggests that whatever the dominant plasma flow in the magnetodisk and plasma sheet is, its electrodynamic coupling to the two hemispheres is partly asymmetric.

### 4.4 Observation of Radio Emissions Above the Main Oval

For a significant part of the 27 crossings studied in this paper, a radio emission is observed close to the local electron cyclotron frequency *f*_ce_, on the equatorward side of the main oval. The radio emission at this frequency (HOM to DAM wavelengths) are produced via the electron Cyclotron Maser Instability mechanism (eCMI), at a frequency close to the local electron cyclotron frequency (at a few percent above it, see Kurth, Imai, et al., 2017; Louarn et al., 2017; Louis et al., 2019). Therefore, if an emission is observed close to the local *f*_ce_, it means that Juno is crossing (or flying very close to) a radio source. The radio source is never observed directly above the brightest UV emission. Compared to the common case studied here and by Mauk et al. (2020), the radio emissions are only observed in the “diffuse aurora” (difA) area, ending at (or just before) the Zone‐I (ZI) area. Quoting Mauk et al. (2020), “the difA zone is characterized by (a) electron populations with electron intensities outside of the loss cone larger than the intensities inside the loss cone and (b) downward intensities and energy fluxes within the downward loss cone greater than the intensities and energy fluxes within the upward loss cone,” while the ZI “is characterized by (a) electron intensities within the downward loss cone greater than the intensities outside of the loss cone and (b) downward intensities and energy fluxes greater than the upward intensities and energy fluxes.” The eCMI radio emissions are believed to be mostly produced by upward electrons (Louarn et al., 2017) through a loss‐cone distribution function (i.e., an empty loss cone that make the distribution unstable), with sometimes the addition of conics (Menietti & Burch, 1985) in the electronic distribution function, seen in both parallel and anti‐parallel directions (i.e., upward and downward propagating waves may be amplified, see Louarn et al., 2018). Therefore, it is not surprising that emissions are observed above the difA (characterized by electron intensity outside the loss cones larger than inside the loss cones in both parallel and anti‐parallel directions) and that no emission is observed above ZI which is characterized by a strong downward electron flux inside the loss cone, and these last observations seem to even confirm the theory of the production of radio emission through the loss cone‐driven eCMI.

## 5 Conclusions

In this study, we applied the multi‐instrument analysis method first described by Wang et al. (2021), to study the first 30 orbits of Juno in order to derive the Magnetosphere‐Ionosphere‐Thermosphere coupling key parameters along Juno's magnetic footprint. It allowed us to demonstrate the applicability of Wang et al.’s method to a much larger data set, and to provide a picture of the relative variations of MIT coupling parameters across the regions of main auroral emission. Studying the orbits of Juno up to the 30th orbit made it possible to extend the range of MLT studied in Wang et al. to a large fraction of the southern and northern afternoon sectors, taking advantage of the azimuthal drift of Juno's orbit with time, and allowed us to extensively study and compare the two conjugate auroral regions.

This systematic study of MIT coupling parameters provided some clues on how ionospheric closure of field‐aligned currents into the two conjugate hemispheres couples the two main oval regions to magnetospheric dynamics, and on the consistency of the corotation enforcement model. Calculated profiles of MIT coupling parameters across the main ovals displayed a large orbit‐to‐orbit and inter‐hemispheric variability, revealing structures extending over small latitudinal spans, which are still to be understood, such as the occurrence of several layers of upward‐downward field‐aligned current pairs distributed in latitude during a single crossing. Yet, statistical analysis allowed us to capture some dominant trends for both hemispheres. In the southern hemisphere, our calculated current systems display a trend consistent with the generation of a region of sub‐corotating ionospheric plasma poleward of the main aurora, in agreement with the corotation enforcement models of Hill (2001), and Cowley and Bunce (2001) and their following publications. Conversely, in the northern hemisphere, current systems of the two opposite trends, producing respectively sub‐corotation consistent with the corotation enforcement model discussed above, and super‐corotation, are observed in the afternoon MLT sector to which our study is limited. This large variability observed from orbit to orbit and between the two conjugate auroral regions demonstrates that our current theoretical understanding of MIT coupling at Jupiter is still limited, and calls for further observations and theoretical studies, particularly on interhemispheric coupling of auroral phenomena, currents and plasma flows.

The larger data set to be provided by the Juno Extended Mission will allow us to move in this direction and provide a better MLT coverage of the main ovals. The study of a few Io auroral tail crossings initiated in this study will also be spectacularly expanded with the Extended Mission, allowing a more systematic study of Galilean moons tails, flux tubes and close plasma environments.

Finally, the method extensively used in this study can be applied to MIT coupling at Saturn, using data from the high‐inclination, F‐ring and Grand Finale orbits, and to other future missions to giant planets, including ice giants, that will offer a similarly favorable orbit geometry with respect to the auroral regions.

## Supporting information

Supporting Information S1

## Data Availability

All Juno data used in this study are available at the Planetary Data System of NASA and can also be accessed through the AMDA (http://amda.cdpp.eu/, Génot et al., 2021) tool developed by CDPP (http://cdpp.eu) and the CLweb tool developed by Emmanuel Penou at IRAP (http://clweb.irap.omp.eu). For cross‐comparisons purposes, the authors made use of the APIS service (https://apis.obspm.fr, Lamy et al., 2015). The scripts used to perform the study are available on GitHub and archived in Zenodo (Al Saati et al., 2022a). For practical purposes, all the data needed to run the codes are archived in Zenodo (Al Saati et al., 2022b).
