# Supplementary material for: Magnetosphere‐Ionosphere‐Thermosphere Coupling Study at Jupiter Based on Juno's First 30 Orbits and Modeling Tools
Source: J Geophys Res Space Phys. 2022 Oct 9;127(10):e2022JA030586. doi: 10.1029/2022JA030586 (PMC9787687; doi:10.1029/2022JA030586)
Supplement: Supplementary file 1 — Supporting Information S1 [file JGRA-127-e2022JA030586-s001.pdf]

Supporting Information for Magnetosphere-Ionosphere-Thermosphere Coupling study at Jupiter Based on Juno's First 30 Orbits and Modelling Tools

S. Al Saati[1,2], **N. Clément**[1,3], **C. Louis**[4,1], **M. Blanc**[1,5], **Y. Wang**[6], **N. André**[1], **L. Lamy**[7,5], **B. Bonfond**[8], **B. Collet**[5], **F. Allegrini**[9], **S. Bolton**[9], **G. Clark**[10], **J. E. P. Connerney**[11], **J.-C. Gérard**[8], **G. R. Gladstone**[9], S. **Kotsiaros**[12], **W. S. Kurth**[13], **B. Mauk**[10]

[1] IRAP, CNRS, Université Toulouse III-Paul Sabatier, CNES, Toulouse, France
[2] CPHT, CNRS, Institut Polytechnique de Paris, Palaiseau, France
[3] Laboratoire d'Astrophysique de Bordeaux, Université de Bordeaux, France
[4] School of Cosmic Physics, DIAS Dunsink Observatory, Dublin Institute for Advanced Studies, Dublin 15, Ireland
[5]LAM, Pythéas, Aix Marseille Université, CNRS, CNES, Marseille, France
[6] State Key Laboratory of Space Weather, National Space Science Center, Chinese Academy of Sciences, Beijing, China
[7] LESIA, Observatoire de Paris, Université PSL, CNRS, Sorbonne Université, Université de Paris, Meudon, France
[8] University of Liège, Belgium
[9] SwRI, San Antonio, TX, USA
[10] JHU-APL, Laurel, MD, USA
[11] NASA-Goddard Space Flight Center, Greenbelt, MD, USA
[12] Technical University of Denmark
[13] University of Iowa, USA

Introduction

In this Supplementary Information document, we provide more details about the numerical methods used in the study described in the main paper. We discuss first in section 1 a correction in the electrodynamics model which allows us to take into consideration the uncertainties in the data to compute the MIT coupling parameters. Secondly, in section 2, we describe the method developed for this study to compute the residual magnetic field from the Juno MAG instrument and the JRM09 magnetic model using Fast Fourier Transform filtering methods. Thirdly, in section 3 we describe briefly the method used to determine the amount of time by which we shift the UVS data. Finally, in section 4, we describe the method used to compute the uncertainties over the calculated MIT coupling parameters.

1 Electrodynamics model implementation

1.1 Reference frames for the electrodynamics model

In our calculation scheme, we perform a geometric mapping of the parameters that are measured by Juno in the 3-D space corresponding to the "high latitude, auroral and polar magnetosphere" region, referred to as Region II in Wang et al. (2021), to the conducting layer of the ionosphere/thermosphere, referred to as Region III, along magnetic field lines. This mapping is done to calculate the key parameters of MIT coupling in this layer, which is modelled as a 2-D infinitely thin shell surrounding the planet. Indeed, its vertical thickness, on the order of 3.10^{-3} times the Jovian radius R_J , is very small compared to the planetary scale. To cover Region II, region III and their connections, we use two different reference frames just as in Wang et al. (2021), and as described in figure 1.

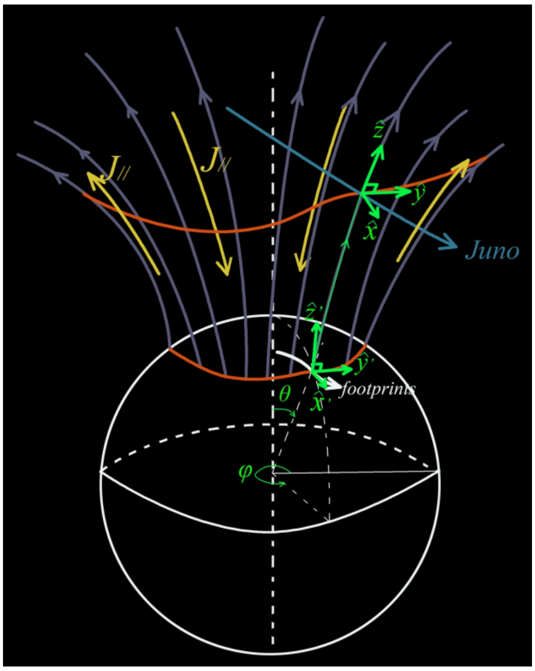

Figure 1: Northern hemisphere illustration of the geometric elements and reference frames used in our calculations of MIT coupling parameters from Juno data. The magnetic field lines mapping to the mean latitude of the main oval (shown as the lower red curve on the ionospheric sphere) are shown in purple. They make it possible to map this main oval location to the altitude of Juno (upper red curve). Juno's trajectory as well as the trajectory of its magnetic footprint are in sky-blue and white, respectively. Two local reference frames are used for our differential calculations. At the altitude of Juno and everywhere along magnetic field lines above the ionosphere, unit vector $\hat{z}$ points along the magnetic field, $\hat{y}$ is positive eastward along the tangent to the cone of field lines connected to the main oval, and $\hat{x}$ complements the frame, positive towards the equator in both hemispheres. At the ionospheric altitude, unit vectors $\hat{x}'$ and $\hat{y}'$ are horizontal and tangent to the conducting layer of the ionosphere which is assumed to be an infinitely thin surface surrounding the plane. $\hat{x}'$ is oriented towards the equator orthogonal to the auroral oval (red curve), $\hat{y}'$ is oriented eastward along the oval, and $\hat{z}'$ is vertical, positive upward in the northern hemisphere and downward in the southern hemisphere. $\hat{x}'$ and $\hat{y}'$ taken together provide a local 2-D reference frame for horizontal vectors and differential expressions defined in the plane tangent to the ionospheric conducting layer. Figure from Supplementary Information of Wang et al. (2021).

For the description of the 3-D space of Region II, we introduce a set of orthogonal curvilinear coordinates (x, y, z) such that x and y are constant along magnetic field lines, to facilitate the mapping of quantities between Juno's location and its magnetic footprint in the ionosphere/thermosphere. Each point in (x, y, z) space is associated with a local orthogonal reference frame with vector units $(\hat{x}, \hat{y}, \hat{z})$. $\hat{z}$ is along the magnetic field direction, $\hat{y}$ is orthogonal to $\hat{z}$ in the plane containing the local magnetic field vector and the local tangent to the oval, and $\hat{x} = \hat{y} \times \hat{z}$. In this reference system, distances are given by the metric tensor $\mathrm{d}s^2 = h_x^2 \mathrm{d}x^2 + h_y^2 \mathrm{d}y^2 + h_z^2 \mathrm{d}z^2$, in which h_x , h_y and h_z are the metric coefficients in the three directions. In the dipolar approximation, where we assume that the magnetic field is radial, we can choose to work in the spherical frame given by $(x, y, z) = (\theta, \phi, r)$. We then have explicitly $h_x = h_\theta = r$, $h_y = h_\phi = r \sin\theta$ and $h_z = h_r = 1$.

To map quantities along field lines, we use the JRM09 magnetic field model of Connerney et al. (2018) complemented by the current sheet model of Connerney et al. (1981). For the description of parameters related to the 2-D space of Region III, we use a reference set (x', y') which defines unit vectors $\hat{x}'$ and $\hat{y}'$. For convenience, we choose this set such as $\hat{x}'$ points horizontally equatorward and orthogonal to the main oval, and $\hat{y}'$ points eastward along the main oval. The parameters defined in this 2-D space of Region III are either scalars (Joule and particle heating), or 2-D vectors tangent to the ionospheric shell (i.e., height-integrated ionospheric currents, horizontal ionospheric electric fields), or 2-D tensors such as the horizontal height-integrated ionospheric conductance tensor. In this reference system, distances are given by the metric tensor $(\mathrm{d}s')^2 = h_{x'}^2 (\mathrm{d}x')^2 + h_{y'}^2 (\mathrm{d}y')^2$, in which $h_{x'}$ and $h_{y'}$ are the metric coefficients in the two horizontal directions. This 2-D set can be complemented by a vertical unit vector pointing z' upwards in the northern hemisphere and downward in the southern hemisphere.

1.2 Assumption of homogeneity of MIT coupling parameters along the direction of the main oval

Calculating the MIT coupling parameters in region III is possible when one can assume that the variations of all quantities along the main auroral oval are much smaller than the variations orthogonal to the oval and to the local direction of the magnetic field, and only in that case. The fulfillment of this condition is checked case by case (i.e., for each North and South perijove separately) by an inspection of UVS brightness images: we use these images to check whether the main oval is reasonably homogeneous along its longitudinal extension and much thinner than its longitudinal extension in the vicinity of its crossing with the Juno ionospheric magnetic footprint. When this condition is fulfilled, one can write $\frac{\partial}{\partial x} \gg \frac{\partial}{\partial y}$ in Region II, and similarly $\frac{\partial}{\partial x'} \gg \frac{\partial}{\partial y'}$ in Region III.

1.3 Calculation of $J_{\|,\mathrm{iono}}$ from the magnetic field perturbation at the altitude of Juno

Using Ampère's law at Juno location under the approximation $\frac{\partial}{\partial x} \gg \frac{\partial}{\partial y}$ provides the following relationship between the magnetic perturbation and $J_{\|,\mathrm{Juno}}$:

$$J_{\|,\mathrm{Juno}} = J_z = \frac{1}{\mu_0 h_x h_y} \frac{\partial \left(h_y \delta B_y\right)}{\partial x} \tag{1}$$

where μ_0 is the vacuum permittivity. The magnetic perturbation δB_y is obtained from the Juno magnetometer measurement based on the method described in section 2 of this Supplementary Information document.. Using the conservation of magnetic flux along each field line

$$\left(\frac{J_\|}{B}\right)_{\mathrm{iono}} = \left(\frac{J_\|}{B}\right)_{\mathrm{Juno}} \tag{2}$$

one can write a direct relationship between the magnetic perturbation generated by field-aligned currents at the altitude of Juno and the intensity of these same currents at the top of the ionosphere

$$J_{\|,\mathrm{iono}} = \frac{1}{\mu_0} \frac{B_{\mathrm{iono}}}{B_{\mathrm{Juno}}} \frac{\partial \left(h_y \delta B_y\right)}{h_x h_y \partial x} \tag{3}$$

1.4 Calculation of J_x from $J_\|$ at the ionospheric altitude

Knowing $J_{\|,\mathrm{iono}}$, a 2-D electric current continuity equation can be obtained for the 2-D horizontal height-integrated ionospheric current $\vec{J} = (J_{x'}, J_{y'})$, in units of A/m, flowing in the ionospheric conductor (Region III) by integrating the 3-D current continuity equation $\nabla \cdot \vec{J} = 0$ vertically across the ionospheric conductor. It reads:

$$\frac{\partial \left(h_{y'} J_{x'}\right)}{h_{y'} h_{x'} \partial x'} = -J_{\|,\mathrm{iono}} |\sin I| \tag{4}$$

where $J_{\parallel,\mathrm{iono}}$ is positive for upward current, and I is the inclination angle of the magnetic field with respect to the local vertical at Juno's ionospheric footprints.

1.5 Simplifying assumptions made in the context of this study

In the specific case where, at least locally, the oval runs along a circle of constant magnetic latitude, one can introduce the global magnetic coordinates such that $(x', y') = (\theta, \phi)$ and extend this coordinate system along each field line to generate the $(\hat{x}, \hat{y}, \hat{z})$ coordinate system of region II. In this particular case, $h_{y'} = r \sin\theta$, $h_{x'} = r$, where r is the radial distance to the planet's center and equations 3 and 4 write respectively:

$$J_{\parallel,\mathrm{iono}} = \frac{1}{\mu_0} \frac{B_{\mathrm{iono}}}{B_{\mathrm{Juno}}} \frac{\partial\left(\sin\theta \delta B_\phi\right)}{r \sin\theta \partial\theta} \tag{5}$$

$$\frac{\partial\left(\sin\theta J_\theta\right)}{\partial\theta} = -r J_{\parallel,\mathrm{iono}} |\sin I| \sin\theta \tag{6}$$

The global reference frame considered in this study is Jupiter's magnetic dipole coordinate system linked to the dipole component of the JRM09 model. What was observed is that for the major part of the crossings considered in this study, the azimuthal direction in the global reference frame was locally approximately coincident with the direction tangent to the main oval. We thus approximated, in the context of this study, the coordinates x' and y' to be respectively the θ and ϕ coordinates of the global magnetic reference frame.

However, the calculations described above using equations 3 and 4 are exact as long as the assumptions $\frac{\partial}{\partial x} \gg \frac{\partial}{\partial y}$ and$\frac{\partial}{\partial x'} \gg \frac{\partial}{\partial y'}$ are correct. These calculations can be rigorously done independently of the choice of the global reference frame, as long as the local reference frames are identified exactly. Moreover, these calculations do not assume any azimuthal symmetry, such that they can be applied to compute the MIT coupling parameters in the Northern hemisphere as well.

Thus, using these simplifying assumptions in the context of this study, $J_{x'}$ can be derived by integration of equation 4 over x' or equivalently J_θ by integration of equation 6 over θ, starting from an appropriate boundary, poleward or equatorward of the main oval, where horizontal ionospheric currents have to be zero, for instance a region where electron precipitation fluxes in our data are negligible, producing near-zero ionospheric conductances. What differs in this calculation from the one described in Appendix A-3 of Wang et al. (2021) is the numerical integration of equation 6 with boundary conditions identified case by case to retrieve the ionosphere current J_θ, rather than integrating formally equation 6 to retrieve equation equation A17 Wang et al. (2021). The choice of computing J_θ from numerical integration was motivated by our analysis of the errors in our results. Rather than computing J_θ over intervals of arbitrary length as a primitive of δB_ϕ, we choose to compute J_θ over restricted intervals of a few degrees of latitude as it was shown in the main paper and as described in section 4 of this Supplementary Information document. This method yields better control over the uncertainties on the results.

Once $J_{x'}$ (or equivalently J_θ) has been calculated, the calculation proceeds as in Appendix A-3 of Wang et al. (2021): ionospheric Pedersen and Hall conductances are calculated from electron precipitation fluxes using the simplified ionospheric model as in Appendix A-2. Then the horizontal electric field $E_{x'}$ or E_θ and the total Joule power dissipation are calculated using the ionospheric Ohm's law and neglecting neutral winds, as discussed in the description of the electrodynamics model in the main paper.

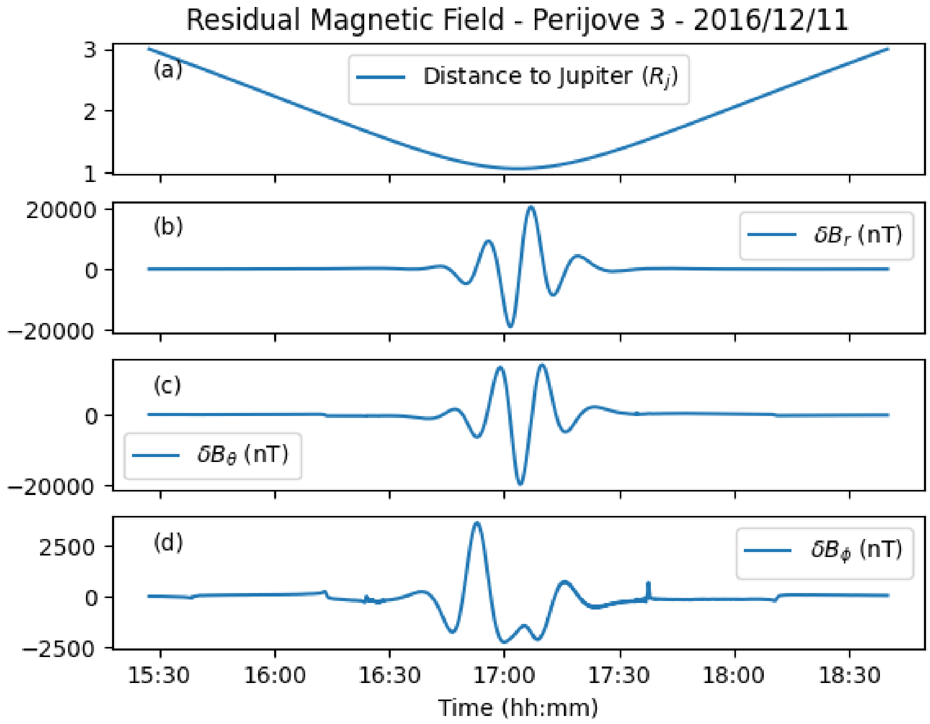

Figure 2: Residual magnetic field calculated according to equation 7 in spherical coordinates for perijove 3 on 11-dec-2016 from 15:30 to 18:30, Juno time. Panel (a): distance of the Juno spacecraft to Jupiter, in Jovian Radii ($R_j = 71.492$ km). Panel (b): Residual radial magnetic field δB_r. Panel (c): Residual polar magnetic field δB_θ. Panel (d): Residual azimuthal magnetic field δB_ϕ. Analysis of this figure shows that the amplitude of the residual magnetic field remains close to zero when Juno is far from Jupiter ($r_{\text{Juno}} > 1.5R_j$). But when Juno approaches Jupiter, the residual magnetic is modulated by oscillations of very large amplitude (tens of mT for δB_r, δB_θ, and few mT for δB_ϕ) and very large period (> 20 min) with respect to the expected amplitude and period of the signal of interest in this study (hundreds of nT for the amplitudes, and few minutes for the period). This observation suggests that filtering away this large amplitude/low frequency component of the signal should give back the signal of interest.

2 Filtering Mag Data

We discuss in this section of the Supplementary Information the method used to compute the residual magnetic field from Juno's MAG instrument data and the JRM09 model magnetic field.

Computing the residual magnetic field $\delta\vec{B}$ for each crossing is an essential step for the derivation of the MIT coupling key parameters. This residual magnetic field is defined as follows:

$$\delta\vec{B} = \vec{B}_{MAG} - \vec{B}_{JRM09+CAN81} \qquad (7)$$

where $\vec{B}_{MAG}$ is the magnetic field measured by Juno's MAG instrument, and $\vec{B}_{JRM09+CAN81}$ is the magnetic field obtained from the JRM09 + CAN81 magnetic field model. Computing this residual magnetic field is the key to the calculation of field-aligned currents at the top of the ionosphere via equation 3.

Inspection of figure 2 shows that the amplitude of the residual magnetic field remains close to zero when Juno is far from Jupiter, but that this residual field is modulated by oscillations of very large amplitude (of the order of tens of mT for δB_r, δB_θ, a few mT for δB_ϕ) and very large period (of the order of 20 min). These amplitudes and period are much larger than the ones expected for the signal of interest in this study, which reach maximal amplitudes of hundreds of nT, and whose period is of a few minutes at most. We thus need to extract from this data the data which interests us, and whose amplitudes and characteristics in the frequency domain we know already. To do this, Kotsiaros et al. (2019) ran a robust splines fit with a resolution of 2 minutes in order to remove such residual trends in the data. This method was also applied by Wang et al. (2021). Here, we propose a similar method using Fast Fourier Transform filtering. This method was first used for investigating large volumes of MAG data in the preliminary phase of the study, but was refined progressively as the study progressed. Initially, we considered a set of about 60 crossings, from which 27 were selected after inspection of the UVS data and the residual magnetic field data. In this context, we proposed this new preprocessing method enabling us to process automatically the identification of the low frequency component of the signal we needed to extract, from the complete signal described in figure 2. In the following, we describe more extensively this method, and the analysis done in order to estimate the intrinsic relative uncertainty on the results calculated from this method.

As we mentioned it, this method consists essentially in a band pass Fast Fourier Transform filtering. Indeed, we identified, at least, three components in the signal:

- a high frequency component corresponding to the rotation of Juno around its axis, with a period 30 seconds.
- a low frequency component corresponding to the error generated by the magnetic model when Juno crosses the perijove region, corresponding to periods of about 1 hour.
- Then, the main component of the signal which is of interest for our study and that we want to extract, corresponding to time variations of the order of 10 minutes.

We thus apply a bandpass FFT filter on the residual magnetic field signal, of upper cutoff frequency f_u and lower cutoff frequency f_l such that $10\text{min} \gg 1/f_u \gg 30\text{s}$ and $1\text{h} \gg 1/f_l \gg 10\text{min}$.

However, such a filtering method intrinsically brings error in the resulting signal, which amounts to introducing "artificial" currents in the system we are considering. These artificial currents can be understood visually from figure 3. We thus needed to quantify the amount of artificial currents we are introducing for each crossing. To do so, the method proposed is the following: we assume first that the azimuthal component of the residual residual magnetic field δB_ϕ is entirely generated by field aligned currents $J_\parallel$, according to equation 3. We thus consider a "dummy" profile of field aligned currents $J_{\parallel,0}$, and from this profile, we compute the resulting δB_{ϕ_0}. We apply the filtering method of this dummy $\delta B_{\phi,0}$, which gives us $\delta B_{\phi,f}$. From this filtered component, we compute back the resulting fields aligned currents $J_{\parallel,f}$, which we compare to the original $J_{\parallel,0}$. This process being linear on its entry signal $J_{\parallel,0}$, it only suffices to realize this study for a well chosen set of entry signals to be able to determine the response of our system for any entry signal of arbitrary complexity, such as the signal measured by Juno for example. Linearity is thus very helpful for this analysis.

From our previous analysis from figure 3, we identify three main input parameters which are relevant for this analysis, namely the time period $T_J = 30\text{s}$ corresponding to Juno's rotation around its axis, the time period $T_1 \approx 2\text{min}$ at which the signal of interest occurs, and the time period $10\text{min} \ll T_0 = 1/f_l \ll 1\text{h}$ corresponding to the lower cutoff frequency of the FFT filter. We then identify three effects of the filtering

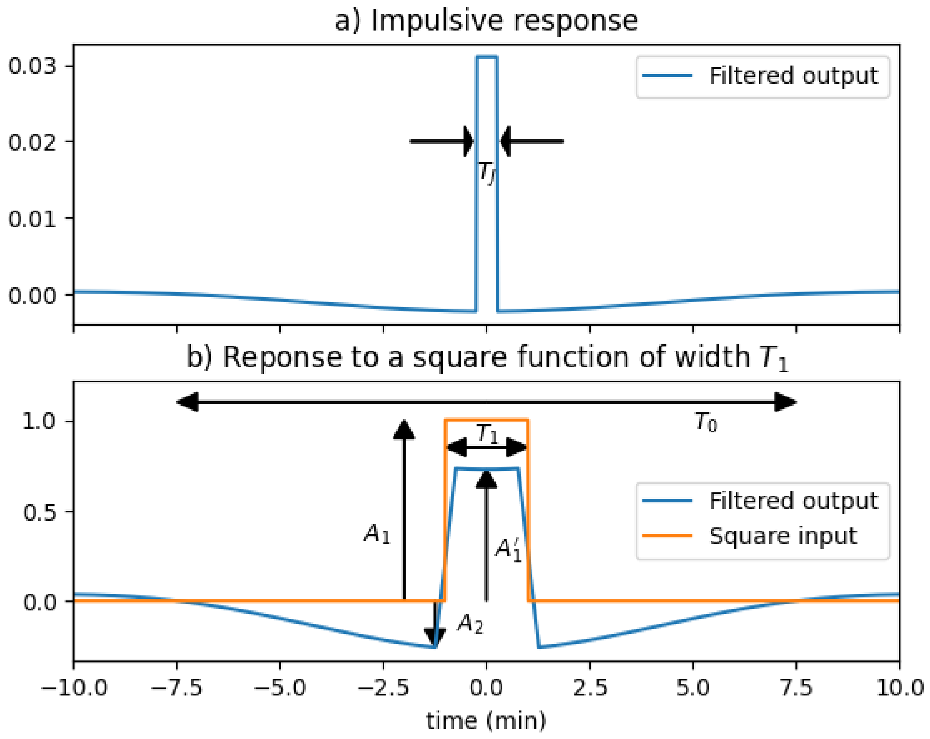

Figure 3: Effects of the filtering method on the calculated field aligned currents, depending on the form of the input. The effects were exaggerated on the figure. Panel a: Response calculated for an impulsive input, showing a characteristic timescale T_J corresponding to Juno's rotation around its axis. Panel b: Response to a square input, where we denoted by A_1 the amplitude and by T_1 the timescale of the input, by T_0 the high characteristic timescale of the applied filter, A_1' the reduced amplitude of the output and A_2 the amplitude of the side currents appearing as a result of the filtering. The aim is to characterize A_1'/A_1 and A_2/A_1 as a function of T_1/T_0 to be able to estimate the error generated by such filtering method for each crossing.

of the signal. The first effect corresponds to the broadening of all the narrow peaks in the entry signal, and this broadening effect is the same for all crossings. The second effect is the lowering of the amplitude of the signal, denoted by A_1 in the input signal and A_1' for the output signal. This effect is entirely characterized by the value of the ratio A_1'/A_1. Then, the third effect is the generation of a negative artificial signal on either sides of the square, with an amplitude A_2. This corresponds to the artificial currents mentioned earlier, and this effect can be characterized by the ratio A_2/A_1. These effects can be estimated once and for all for all crossings by computing the quantities A_1'/A_1 and A_2/A_1 as functions of the ratio T_1/T_0, as shown in figure 4. For each crossing, it suffices then to compute the value of the ratio T_1/T_0 given by the signal and the frequency of the applied filter to estimate precisely the error introduced by this filtering method.

This analysis allowed us the estimate systematically the error introduced by this filtering method for each crossing. The choice of the value of lower cutoff frequency $1/f_l$ of the FFT filter was chosen differently for each crossing so as to minimize both the error introduced by this method and the error coming from the JRM09 model, depending on the quality of the data which was available for each crossing. Overall, choosing an adequate value of f_l with respect to the data was possible, but in some extreme cases,

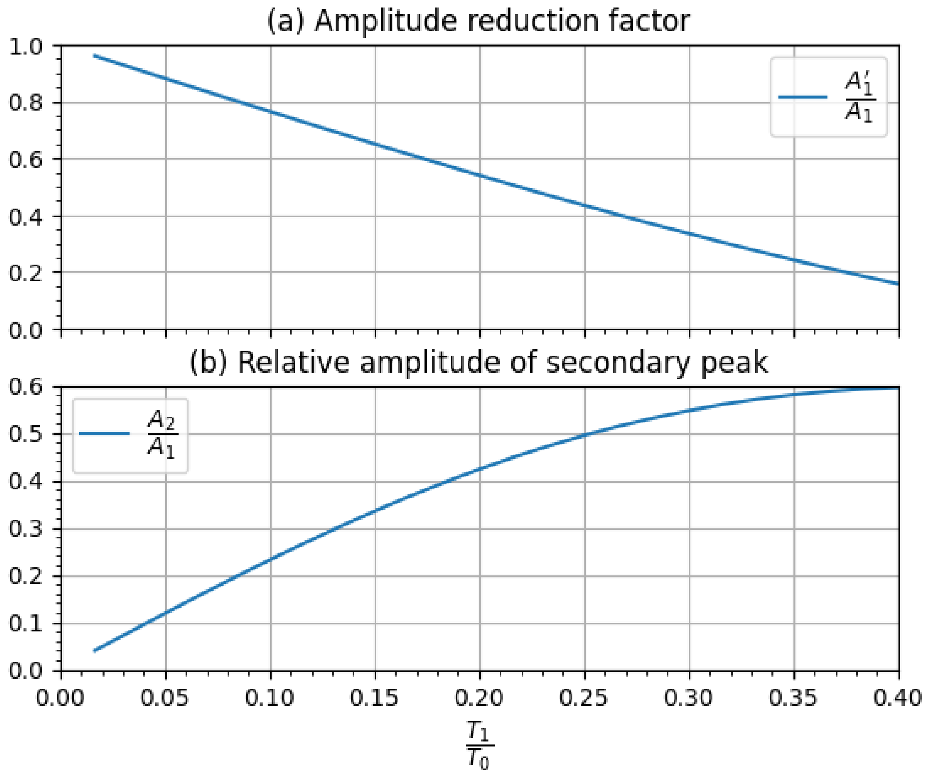

Figure 4: Diagram describing the amplitude reduction factor A_1'/A_1 (panel a) and the relative amplitude of the secondary peak A_2/A_1 (panel b) as a function of the ratio T_1/T_0. This diagram allows for a systematic estimation of the error generated by the filtering method on the resulting MIT coupling parameters.

we had to choose larger values of the ratio $T_1/T_0 = T_1 f_l$, so that considering these extreme cases only, we can estimate the error on the calculated field aligned currents to be at most 20%.

3 UVS data time shift

We consider in this section of the Supplementary Information the post processing steps applied on the time series obtained from the UVS instrument data.

During this study, we worked with two kinds of data coming from the UVS instrument: imagery data, and temporal data. The imagery data were used to study visually the homogeneity conditions in the aurora, and allowed for the selection of the crossings studied in the main paper (see for example the UVS maps in figures 3 and 4 of the main paper). Then, the temporal data correspond to the brightness profiles measured by the UVS instrument (Gladstone et al., 2014) along Juno's trajectory. These temporal data are obtained from a given UVS map by reporting the trajectory of the footprint of Juno on this map and by mapping in time the position of the footprint with the brightness measured in this position in the map.

In this paper, the temporal data were compared to the data coming from other Juno instruments, mainly the particle data coming from the JADE and JEDI instruments, and the magnetic data coming from the MAG instrument. We observed on almost all cross-

Crossing	Delay (min:s)	Calculated correlation after delay correction
PJ3N	01:20	26%
PJ5S	00:56	56%
PJ6S	00:46	67%
PJ12N	00:50	55%
PJ22N	01:08	46%

Table 1: Tabular delay and correlation

ings a consistent delay of temporal UVS data with respect to the other Juno instruments. An extensive (visual) analysis showed small fluctuations of this delay depending on the crossing, with an average delay between 50 seconds and 1 minute. Such a delay has also been in other publications (Allegrini et al., 2020), and is believed to originate from uncertainties on the positions of the footprint of Juno on the UVS maps. It should be noted that what appears as a delay when we compare the time series would then actually correspond to a spatial shift of the Juno footpoint on the planet. As a consequence, strictly speaking, we should keep in mind that this shift is not necessarily only aligned with Juno's footpath, but may also be oriented sideways. However, we chose to neglect this effect because we only selected crossing almost perpendicular to the auroral oval and for which the brightness along the oval is relatively constant. Hence this assumption should not affect our conclusions.

To quantify systematically this apparent delay, we assumed that the precipitating particle heating rate computed directly for the JADE and JEDI instruments had to be correlated to the brightness profile from the UVS data. Our method for each crossing then consisted in shifting progressively the UVS data in time by steps of one second, and computing at each step the correlation between the particle heating rate corresponding to the downward electron energy flux in the loss cone measured by the particle detectors and the brightness profile. Then, the shift which maximizes the correlation is retained as the value of the delay for this crossing, and the results are shown in table 1 for the crossings studied in details in the main paper for which UVS data were available. These results show value of delays which are consistent with visual observations. In table 1, we also showed the maximal value of the correlation for its informative value. This correlation seems to reach relevant values for PJ05S and PJ06S reaching nearly 60%, but seems less relevant for PJ03N and PJ22N with values around 20%. However, these low values of the maximal correlation, the delays are in accordance to visual observations.

This method used to quantify the observed delay is by no means an attempt to study comprehensively this issue, as such an attempt from our behalf would be pointless. This method was merely considered as it seemed to provide quantitative arguments about this delay that may be used for further investigations.

4 Uncertainty estimation

To discuss the uncertainties associated with the derivation of the MIT coupling parameters, we are guided by the order at which the calculations are made.

The first parameters derived are the ionospheric conductances Σ_H and Σ_H and the particle heating rate P_e using JADE and JEDI data. The uncertainty here is directly related to the accuracy of the Juno instruments data, which we consider as exact. This

means that if Y is a quantity measured by JADE or JEDI, then the relative uncertainty over this quantity is taken to be zero: $\Delta Y/Y = 0$. The relative uncertainty over the conductances is also directly related to the atmospheric and ionospheric models described in figure 1 of the main paper. A previous analysis conducted by Wang et al. (2021) estimated the relative uncertainty of these models at 20% in the worst case, meaning that if Y is a parameter calculated from either the atmospheric or the ionospheric models, then the relative uncertainty over this parameter is taken to be $\Delta Y/Y = 0.2$. The MIT coupling parameters calculated directly from these modeles are the Ionospheric Pedersen and Hall conductances $\Sigma_{P,H}$, and the particle heating rate P_e. This sets their relative uncertainty as $\Delta\Sigma_P/\Sigma_P = \Delta\Sigma_H/\Sigma_H = \Delta P_e/P_e = 0.2$. Thus, this uncertainty originates from the models.

In parallel, the field aligned currents $J_\parallel$ are computed directly from the residual magnetic field δB_ϕ using the method described in section 2 of the Supplementary Information document. The analysis conducted there allowed us to bound the relative uncertainty associated to $J_\parallel$ to 20% in the worst case, which means that we set $\Delta J_\parallel/J_\parallel = 0.2$. This value is set to take into account the worst cases, which concern only a few crossings of all the crossings selected for this study. For all the other crossings, the relative uncertainty is generally much lower. Another source of uncertainty for $J_\parallel$ could have come from the comparison of the variations observed in the direction along main oval, relatively to the variations observed in the direction perpendicular to the main oval. This comparison is given by the amplitude of $\frac{\partial/\partial y}{\partial/\partial x}$. This parameter is assumed to be small by assumption and will be neglected here.

Then, the ionospheric currents J_x are computed by integration of of equation 6 as was described in section 1.3 of this supplementary information document. The relative uncertainty over J_x evolves then as $\Delta J_x/J_x \propto \Delta\theta \cdot \Delta J_\parallel/J_\parallel$, where $\Delta\theta = \theta_{\text{integration, max}} - \theta_{\text{integration, min}}$ the range over which the integration is computed, from $\theta_{\text{integration, min}}$ to $\theta_{\text{integration, max}}$. The relative uncertainty over $J_\parallel$ is thus accumulated along the computation of the integral, and is amplified by a factor proportional to $\Delta\theta$. Computing J_x over regions of range $\Delta\theta$ arbitrary large will thus result in arbitrary large uncertainties over $\Delta J_x/J_x$. Consequently, the choice made to restrict the computation of J_x over ranges of latitude $\Delta\theta$ such that we always satisfy $\Delta J_x/J_x \leq 0.2$. This impose restricted values for the range $\Delta\theta$, which explains why the parameters plotted in panels (g) to (i) in figures 3, 4 and 5 of the main paper are not calculated for all values of colatitude θ.

Finally, from Appendix A-3 of Wang et al. (2021), we have $E\times B \propto J_x/\Sigma_P$ and $P_J \propto J_x/\Sigma_P$ where $E\times B$ is the azimuthal drift velocity, P_J is the Joule heating rate, J_x the ionospheric current and Σ_P the ionospheric Pedersen conductance. We thus have $\Delta P_J/P_J = \Delta(E\times B)/(E\times B) = \Delta J_x/J_x + \Delta\Sigma_P/\Sigma_P \leq 0.2 + 0.2 = 0.4$. We thus bound from above the relative uncertainties over $E\times B$ and P_J at 40% on the intervals on which these parameters are calculated.
